# PSMA-targeted radiotheranostics in modern nuclear medicine: then, now, and what of the future?

**DOI:** 10.7150/thno.92612

**Published:** 2024-05-13

**Authors:** Mohamed Sallam, Nam-Trung Nguyen, Frank Sainsbury, Nobuo Kimizuka, Serge Muyldermans, Martina Benešová-Schäfer

**Affiliations:** 1 Queensland Micro- and Nanotechnology Centre (QMNC), Griffith University, Nathan Campus, Nathan, QLD 4111, Australia.; 2 School of Environment and Science (ESC), Griffith University, Nathan Campus, Nathan, QLD 4111, Australia.; 3 Griffith Institute for Drug Discovery (GRIDD), Griffith University, Nathan Campus, Nathan, QLD 4111, Australia.; 4 Department of Applied Chemistry, Graduate School of Engineering, Kyushu University, 744 Moto-oka, Nishi-ku, Fukuoka 819-0395, Japan.; 5 Center for Molecular Systems (CMS), Kyushu University, 744 Moto-oka, Nishi-ku, Fukuoka 819-0395, Japan.; 6 Research Center for Negative Emissions Technologies (K-NETs), Kyushu University, 744 Moto-oka, Nishi-ku, Fukuoka 819-0395, Japan.; 7 Laboratory of Cellular and Molecular Immunology (CMIM), Vrije Universiteit Brussel, 1050 Brussels, Belgium.; 8 Research Group Molecular Biology of Systemic Radiotherapy, German Cancer Research Center (DKFZ), Im Neuenheimer Feld 280, 69120 Heidelberg, Germany.

**Keywords:** Prostate cancer, Metastatic castration-resistant prostate cancer, Metastatic hormone-sensitive prostate cancer, Prostate-specific membrane antigen, PSMA-targeted theranostics, Radiotheranostics, Nanoparticles, Antibodies, Inhibitors, Nuclear medicine

## Abstract

In 1853, the perception of prostate cancer (PCa) as a rare ailment prevailed, was described by the eminent Londoner surgeon John Adams. Rapidly forward to 2018, the landscape dramatically altered. Currently, men face a one-in-nine lifetime risk of PCa, accentuated by improved diagnostic methods and an ageing population. With more than three million men in the United States alone grappling with this disease, the overall risk of succumbing to stands at one in 39. The intricate clinical and biological diversity of PCa poses serious challenges in terms of imaging, ongoing monitoring, and disease management. In the field of theranostics, diagnostic and therapeutic approaches that harmoniously merge targeted imaging with treatments are integrated. A pivotal player in this arena is radiotheranostics, employing radionuclides for both imaging and therapy, with prostate-specific membrane antigen (PSMA) at the forefront. Clinical milestones have been reached, including FDA- and/or EMA-approved PSMA-targeted radiodiagnostic agents, such as [^18^F]DCFPyL (PYLARIFY^®^, Lantheus Holdings), [^18^F]rhPSMA-7.3 (POSLUMA^®^, Blue Earth Diagnostics) and [^68^Ga]Ga-PSMA-11 (Locametz^®^, Novartis/ ILLUCCIX^®^, Telix Pharmaceuticals), as well as PSMA-targeted radiotherapeutic agents, such as [^177^Lu]Lu-PSMA-617 (Pluvicto^®^, Novartis). Concurrently, ligand-drug and immune therapies designed to target PSMA are being advanced through rigorous preclinical research and clinical trials. This review delves into the annals of PSMA-targeted radiotheranostics, exploring its historical evolution as a signature molecule in PCa management. We scrutinise its clinical ramifications, acknowledge its limitations, and peer into the avenues that need further exploration. In the crucible of scientific inquiry, we aim to illuminate the path toward a future where the enigma of PCa is deciphered and where its menace is met with precise and effective countermeasures. In the following sections, we discuss the intriguing terrain of PCa radiotheranostics through the lens of PSMA, with the fervent hope of advancing our understanding and enhancing clinical practice.

## Prostate Cancer: The Prologue

Prostate cancer (PCa) is a virtually incurable condition if not diagnosed and treated at the early stage. It is considered the second leading cause of death among men after heart disease 1, 2. The history of PCa, since its discovery more than 200 years ago, is highly diverse, ranging from indolent and slow-growing tumours to overly aggressive histotypes. In 114 nations, PCa had the highest incidence of all male cancers, and in 56 countries, it was the leading cause of cancer-related deaths among men. Currently, PCa remains a significant global public health concern, where three million new incidents of prostate cancer and 416 thousand deaths were reported in 2017 by the Global Burden of Disease (GBD) research. Furthermore, in 2019, the Centers for Disease Control and Prevention (CDC) reported 175 thousand new incidents of PCa and more than 30 thousand fatalities attributable to PCa. Globally, PCa was responsible for more than seven million incapacity-altered lifespans, where these life expectancy durations comprised 88% of the years of defence and approximately 12% of these years of life lived with a disability.

**Figure 1:**
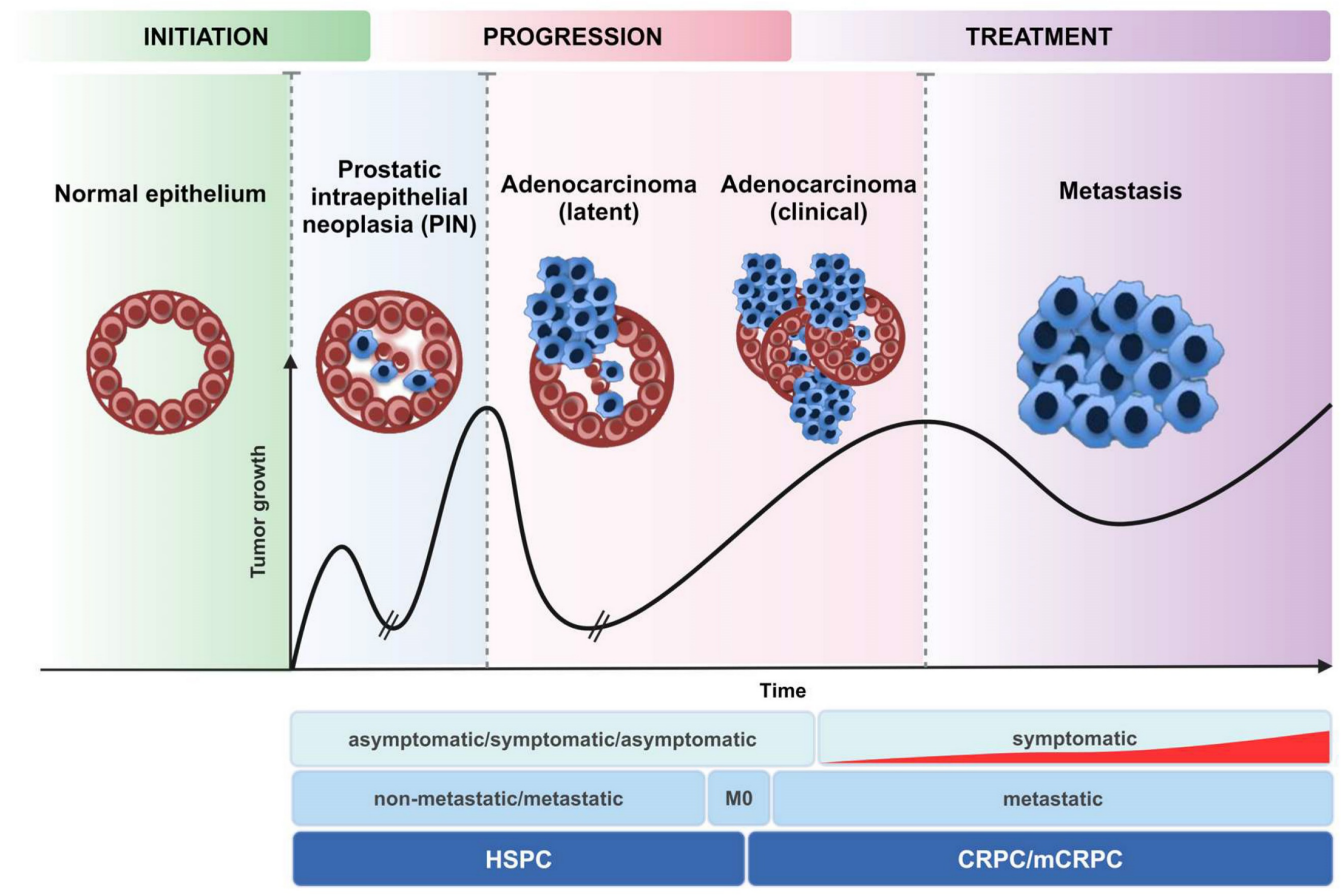
Development of castration-resistant prostate cancer (CRPC) from hormone-sensitive prostate cancer (HSPC). The progression of CRPC is shown as a function of time by plotting an arbitrary tumour volume (ordinate) (arbitrary units). 28% of HSPC patients are diagnosed with CRPC.

Active surveillance is a viable monitoring approach for low-risk individuals with primary PCa. The available focal treatments for those patients include brachytherapy, external beam radiation therapy, and surgical excision; notably, at this stage, all of which are often curative. Patients in the early stages of the disease have a five-year survival rate of greater than 90%. In contrast, patients with advanced PCa whose tumour cells have spread outside the prostate have an inferior quality of life and a 30% likelihood of five-years survival following diagnosis. Androgen deprivation and bone lesion-targeting drugs are some of the most prevalent treatments for advanced PCa. Notably, hormone-sensitive prostate cancer (HSPC) unavoidably advances to castration-resistant prostate cancer (CRPC) due to a variety of resistance mechanisms within cancer cells, such as human androgen receptor variants (hARVs). Metastatic castration-resistant prostate cancer (mCRPC) is a type of PCa that continues to grow even if the amount of testosterone in the body is reduced to very low levels. The mCRPC syndrome is known to be a persistent disease syndrome that can range from asymptomatic to severe debilitating symptoms due to bone or visceral metastasis, even if it is treated with a combination of drugs that suppress secreted antigens and inhibit blood circulation 3. Chemotherapy has played a crucial role for mCRPC patients since the discovery of docetaxel-based therapy in 2004, which has led to an improvement in survival rates. Currently, treatments for mCRPC include therapeutics that target the resistance cascades that lead to CRPC, for instance, abiraterone and enzalutamide, as well as systemic chemotherapies, including docetaxel and cabazitaxel (**Figure 1**) 4. However, despite the advancements made possible by these standard chemotherapy protocols, the gains in survival rates are still inadequate, with cancer cells rapidly developing resistance to these treatment strategies. Therefore, research into PCa continues to focus on elucidating the mechanisms through which cancer cells acquire resistance to chemotherapy and thus creates new therapeutics and possibly synergistic combinations that work more efficiently and help patients live longer 5.

The use of radiolabelled ligands, which identify PCa with great specificity, sensitivity and precisely ablate its location, is a promising new technique for combating this debilitating disease on more individualized basis.

In the following sections, attempts to deliver radiotracers and radiopharmaceuticals to over-expressed extracellular glycoprotein on the surface of PCa cells, mainly prostate-specific membrane antigen (PSMA), will be described. Furthermore, the current options and methods used to detect, define, and treat diseases in this expanding clinical landscape will be discussed. Finally, future strategies for PSMA-based targeted imaging and personalised radionuclide therapy will be explored.

## PSMA: Initiation and Perspective on the Past

Four years after retrieving the androgen-sensitive human prostate adenocarcinoma cell line LNCaP and discovering PSMA in 1983 6, Horoszewicz and colleagues extracted the monoclonal antibody (mAb) 7E11 from LNCaP-immunised mouse hybridomas. Such mAb exhibited a high degree of specificity for both benign and malignant prostatic epithelial membranes. The membrane glycoprotein was designated PSMA since the mAb exhibited no interaction with normal tissues of other investigated body organs 7.

## PSMA Expression and Function in Normal and Malignant Tissues

PSMA expression is approximately a thousand times greater in PCa tissue than in normal prostate tissue, and it is most remarkable in poorly differentiated, castration-resistant tumour cells. It was noted that there was a three- to ten-fold decrease in PSMA expression in the presence of androgens 8. According to the present study, PSMA overexpression in primary prostate tumours increases with tumour grade and the presence of metastatic disease. It was also observed that higher levels of this glycoprotein independently predict worse clinical outcomes.

**Figure 2:**
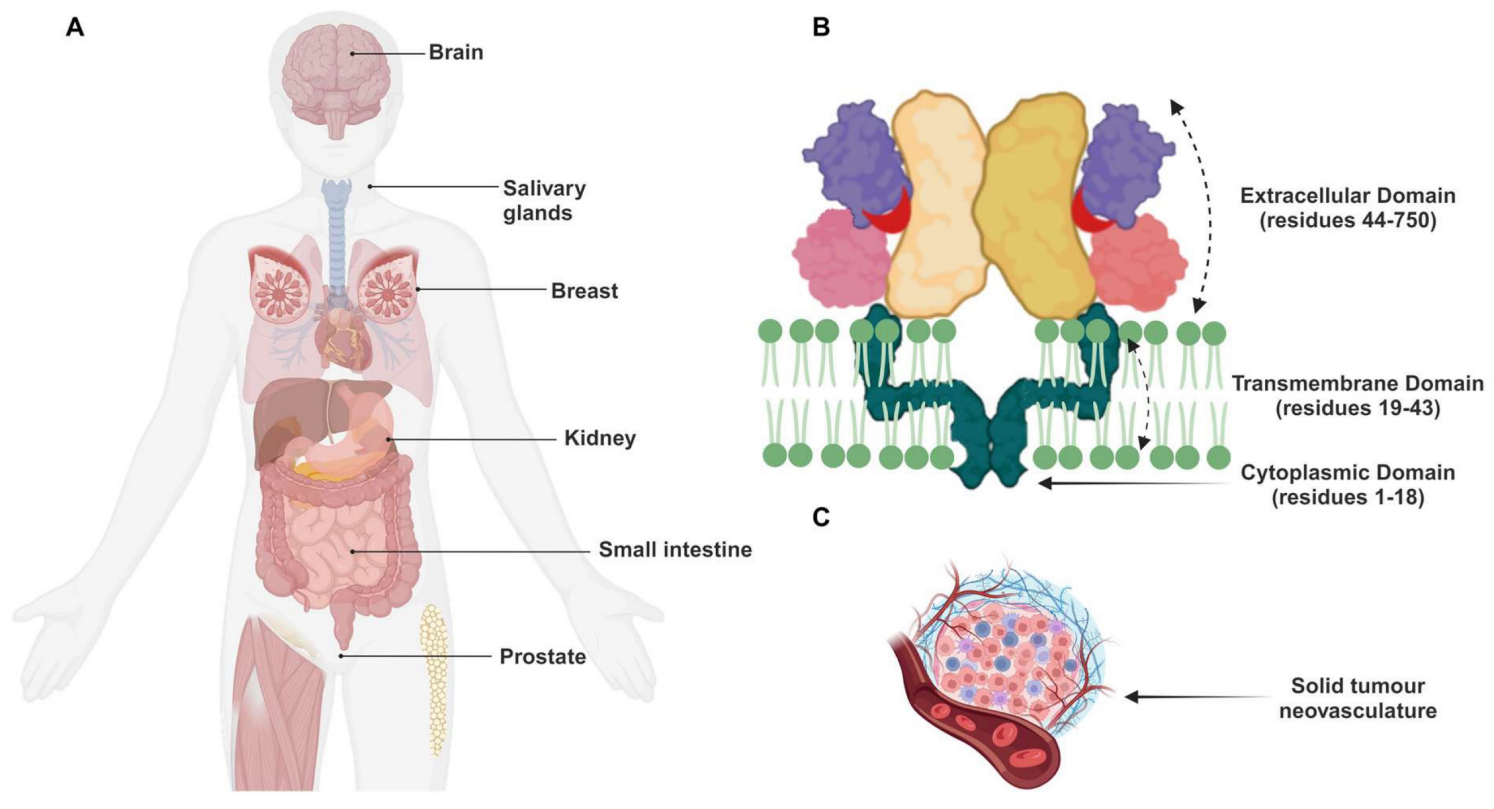
A visual representation of the different components and structures involved in the study of these transmembrane proteins and tumour biology. Illustration showing (**A**) the various expression sites of the GCPII transmembrane protein, (**B**) the composition of the transmembrane protein PSMA, and (**C**) the solid tumour neovasculature. **Figure 2B:** Reproduced with permission from Springer Nature publisher 195.

PSMA is expressed at low levels in the proximal tubules of the kidneys, peripheral ganglia, brain tissues, breast tissue, salivary (parotid, submandibular, sublingual) glands, lacrimal glands, and the intestinal striated border membrane (**Figure 2A**) 9. PSMA is a type II integral membrane glycoprotein that exists on the apical surface of cells as a monomer or homodimer 10. The protein's structure is very similar to that of the human transferrin and consists of a 707-amino acid glycosylated extracellular *C*-terminal region, a 25-amino acid transmembrane domain, and an 18-amino acid cytoplasmic *N*-terminal region (**Figure 2B**) 11.

Before PSMA was recognised and linked to PCa, it was known for its *N*-acetylated alpha-linked acidic dipeptidase (NAALADase) activity in the brain 12. PSMA catalyses the hydrolysis of *N*-acetyl-aspartyl-glutamate (NAAG) into glutamate and *N*-acetyl aspartate (NAA) and contributes to the metabolism of folate and glutamate in certain tissues; thus, PSMA is also known as folate hydrolase 1 (FOLH1) and glutamate carboxypeptidase II (GCPII). For these reasons, PSMA-expressing PCa cells significantly enhance folate uptake and thus grow at substantially accelerated rates 13. Furthermore, the glutamate released from the hydrolase activity of PSMA activates the phospholipase C signalling pathway and promotes tumour growth 14. GPCII hydrolysis of NAAG is a key source of glutamate in late-stage PCa and thus hinders the activity of GPCII *in vivo,* resulting in a reduction in glutamate levels and slowing tumour progression 15. Notably, the amino acid glutamine is essential for the metabolism of rapidly replicating cells. During malignant transformation, glutamine consumption and processing are altered in cancer cells to sustain cell growth and proliferation. In rare instances, cancer cells develop an addiction to glutamine 16.

The PSMA was also lately found on the neovascular endothelium of a variety of tumour types, including renal cell carcinoma, melanoma, colon adenocarcinoma, and lung cancer. However, imaging investigations in humans confirmed that it was not detected in normal endothelial cells 17 18. Furthermore, through its active engagement in the tumour neovascular endothelium, PSMA is believed to contribute to interactions with integrins and endothelial activation. Throughout these events, pro-angiogenic peptides are generated through PSMA-mediated laminin proteolysis 19. In the past four decades, a variety of treatments have been developed to target PSMA after being found to be highly specific for PCa. The PCa-specific diagnostic and therapeutic methods explore low-molecular-weight inhibitors, peptides/peptidomimetics, homo- and heterodimeric ligands, antibodies, antibody fragments, aptamers, and nanoparticles. The clinical findings and implications of the most relevant techniques are discussed in the following sections.

## Radiotheranostics: Tripartite Scheme

**Figure 3:**
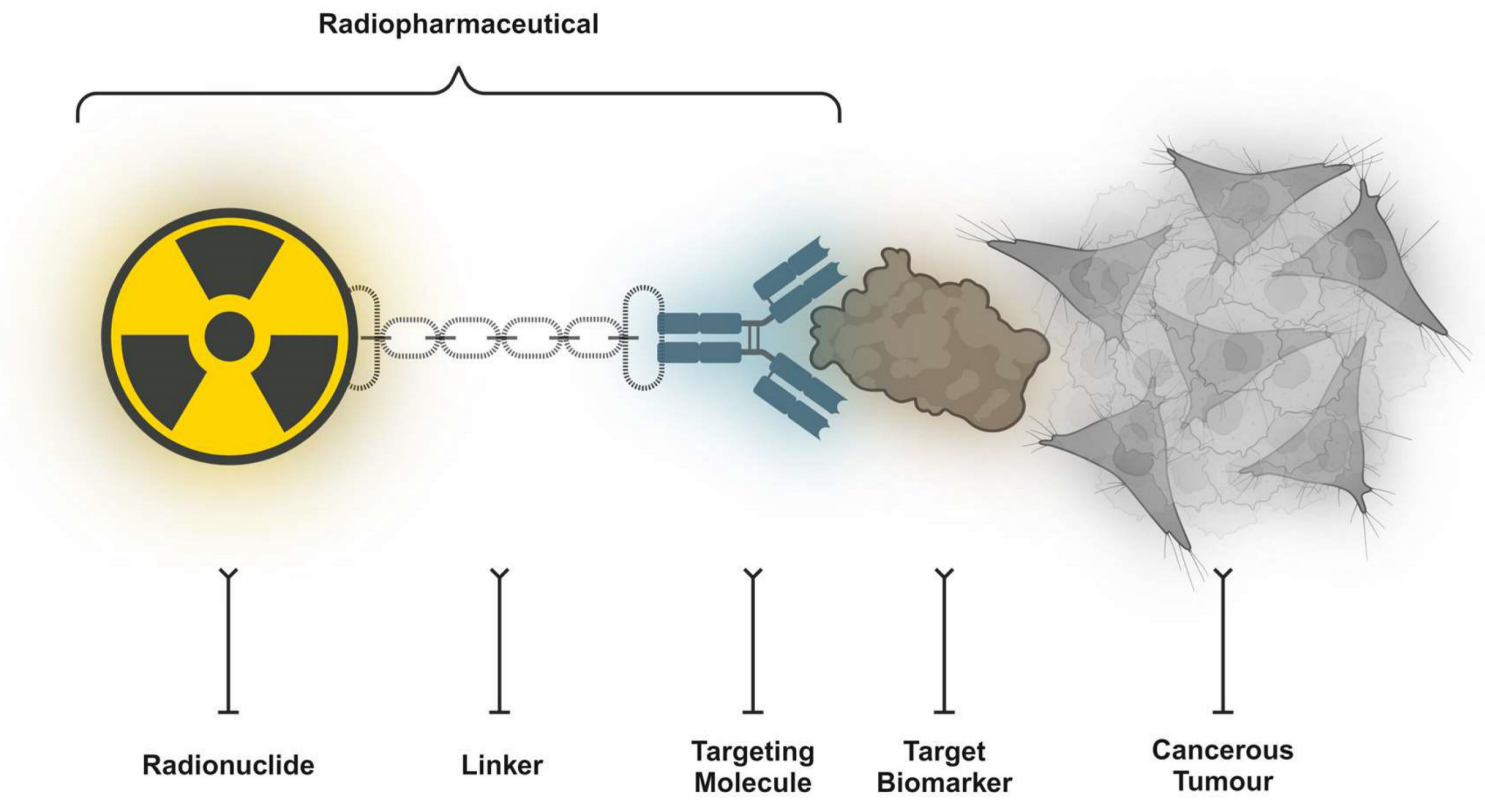
Key components of a conventional PSMA-targeting radiopharmaceutical drug candidate include radiolabelled PSMA-binding domains, linkers, and chelators.

Innovative ideas, new paradigms, and new viewpoints are related to the advancement of medicine. These concepts were developed following the more generalised discoveries of the medical cosmogeny 20. Theranostics is a crucial precision medicine component in nuclear medicine where the three primary components of a radiopharmaceutical agent are (i) a radionuclide (which has diagnostic and/or therapeutic properties), (ii) a chelator/leaving group (which enables the attachment of the radionuclide to the vector), and (iii) a vector (which targets a cancer-specific structure on the surface of the tumour cell with a high affinity) (**Figure 3**). On occasion, a radionuclide can operate as a targeting radioligand; notably, fluorine-18 (^18^F) or radium-223 (^223^Ra). The clearest definition of a “theranostic pair” is one probe labelled with a chemically and physically identical diagnostic or therapeutic radionuclide (or nearly similar). The diminishing similarity between the carried diagnostic and therapeutic compounds might negatively impact the receptor binding affinity. In addition, the biodistribution, pharmacokinetics, and side effects occurrence might alter to some extent as well. The “ideal” theranostic pair ultimately consists of two radioisotopes of the identical element. Radioiodine is a noteworthy example of this method, with iodine-123 (^123^I, single-photon emitter), iodine-125 (^125^I, gamma emitter), or iodine-124 (^124^I, positron emitter) used for diagnostic purposes and iodine-131 (^131^I, gamma and beta minus emitter) utilised for the scintigraphy and treatment of thyroid disorders.

The main differences between these radioisotopes, which dictate their specific application, are related to emission arts, energies and half-lives. The diagnostic equivalent can be achieved using a multitude of combined modalities; for example, concurrent implementation of single-photon emission computed tomography with computed tomography (SPECT/CT), positron emission tomography with CT (PET/CT), or PET with magnetic resonance imaging (PET/MRI), which could result in more sophisticated scans. In the case of SPECT, the chosen radiopharmaceutical is a gamma emitter, while for PET, it is a positron emitter. Due to their exposure limitations, both gamma- and positron-emitting radiopharmaceuticals exhibit high tissue absorption, low energy transfer, and a broad radiation spectrum. In an ideal imaging environment, the patient's radiation exposure should be minimal.

In contrast to other imaging techniques, such as CT and MRI, molecular imaging identifies cancer tissue, function, and biology, which allows for disease localisation, staging and restaging. A unique characteristic of radiotheranostics is the capacity to successfully select patients for subsequent targeted radionuclide therapy (TRNT) based on their likelihood of a positive response to a particular treatment. For the best pre- and post-therapy practice, molecular imaging is performed before treatment to reveal whether the molecular target is adequately expressed by comparing the uptake of radiodiagnostic agent in tumour tissues to that in healthy tissues, which indicates how useful TRNT is for this patient. In addition, these imaging techniques can provide a wealth of information during post-therapeutic follow-up 21, as they can visualize how the patient responded to the treatment 22; additionally, they ease customised dosages (i.e., dosimetry estimation) 23. This theory states that TRNT with the analogical ligand can produce a radiation dose that is predominantly lethal to cancer cells 24. Ionising radiation can induce DNA fragmentation and consequent apoptotic cell death. Subsequently, the ideal radionuclide must be chosen since linear energy transfer (LET) to the target cell affects the degree of cell damage and treatment efficacy, where LET is the transmission rate of energy per unit of track length (keV/µm). Additionally, the periphery of the irradiated tissue region correspondingly expands with the tissue penetration range, which is usually measured in microns and up to several millimetres.

To prolong the therapeutic impact of radiotherapeutic agents, it is preferable to use a radionuclide that has a long half-life (spanning a few days to approximately one or two weeks). Beta minus emitters such as ^131^I, lutetium-177 (^177^Lu), samarium-153 (^153^Sm), holmium-166 (^166^Ho) and yttrium-90 (^90^Y) are the most often employed therapeutic radionuclides in clinical settings because of their half-life and general physicochemical properties (**Table 1**). Beta minus emitters have a LET of 0.1-2 keV/μm and a reasonable tissue range, which spares surrounding non-targeted tissue, but also enables cross-fire effect to some extend 25. Notably, although they share a relatively similar LET range, they exhibit distinct treatment and diagnostic criteria. ^177^Lu, for example, has a substantially lower energy than ^90^Y. Furthermore, ^90^Y is a pure beta-minus emitter, whereas ^177^Lu emits also gamma rays which are suited for SPECT imaging. In comparison to ^177^Lu, ^90^Y is quite penetrant.

In recent years, alpha particle-emitting radionuclides gained a substantial importance in TRNT. The most prominent example is related to the FDA- and EMA-approved radium-223 dichloride (^223^RaCl_2_, Xofigo^®^, Bayer), while other alpha emitters, such as actinium-225 (^225^Ac) and thorium-227 (^227^Th), are the subject of active investigations in preclinical and clinical studies (**Table 2**). Alpha emitters have a very high LET (50-300 keV/μm) and a short tissue range of up to 100 µm which is advantageous for sparing adjacent healthy tissue cells.

Auger electron emitters represent a third class of radionuclides applied in TRNT. These radionuclides are characterised by a high LET (4-26 keV/μm) and a shortest tissue range (<1 µm). Since energy is delivered over such a small distance, Auger electron emitters are particularly efficient intracellularly. If being close to the DNA (for instance, because of cell-penetrating structures or nuclear localisation sequences), they represent an especially promising tool in single cells or microscopic metastases.

^123^I, indium-111 (^111^In), gallium-67 (^67^Ga), and technetium-99m (^99m^Tc) are Auger electron emitters that are being employed for SPECT/CT at extremely low diagnostic dosages 26. However, several of these agents, such as ^123^I, ^111^In, and terbium-161 (^161^Tb), could be administered in large doses to treat thyroid disorders and neuroendocrine tumours (NETs).

Radionuclides typically emit multiple forms of radiation with distinct energy maxima, and some therapeutic radioisotopes can be utilised for non-diagnostic imaging because of this feature. To determine the feasibility of the treatment and rule out pharmacological interference, such non-diagnostic imaging can be very helpful in gathering post-treatment SPECT/CT images 27. This is often the case with beta minus emitters, which might be suitable for SPECT/CT imaging after therapy since they contain a large concentration of gamma emissions. The amount of radiation absorbed by the tumour and healthy tissues can be measured using these images, a process known as dosimetry.

Recent advances in PSMA-targeted radiotheranostics offer the potential to improve the treatment of primary, biochemically recurring, and metastatic PCa. From the perspective of nuclear medicine, a vision for the multidisciplinary applications of PSMA-based approaches is presented. The current and potential consequences for the management of PCa, from early localised to advanced treatment-resistant disease, are explored below while discussing the scientific potential of PSMA-targeted radiotheranostics, as well as the importance of interdisciplinary collaboration in this sector 28.

### PSMA-targeted Radiotheranostics: From Antibodies to Low-molecular-weight Ligands

**Figure 4:**
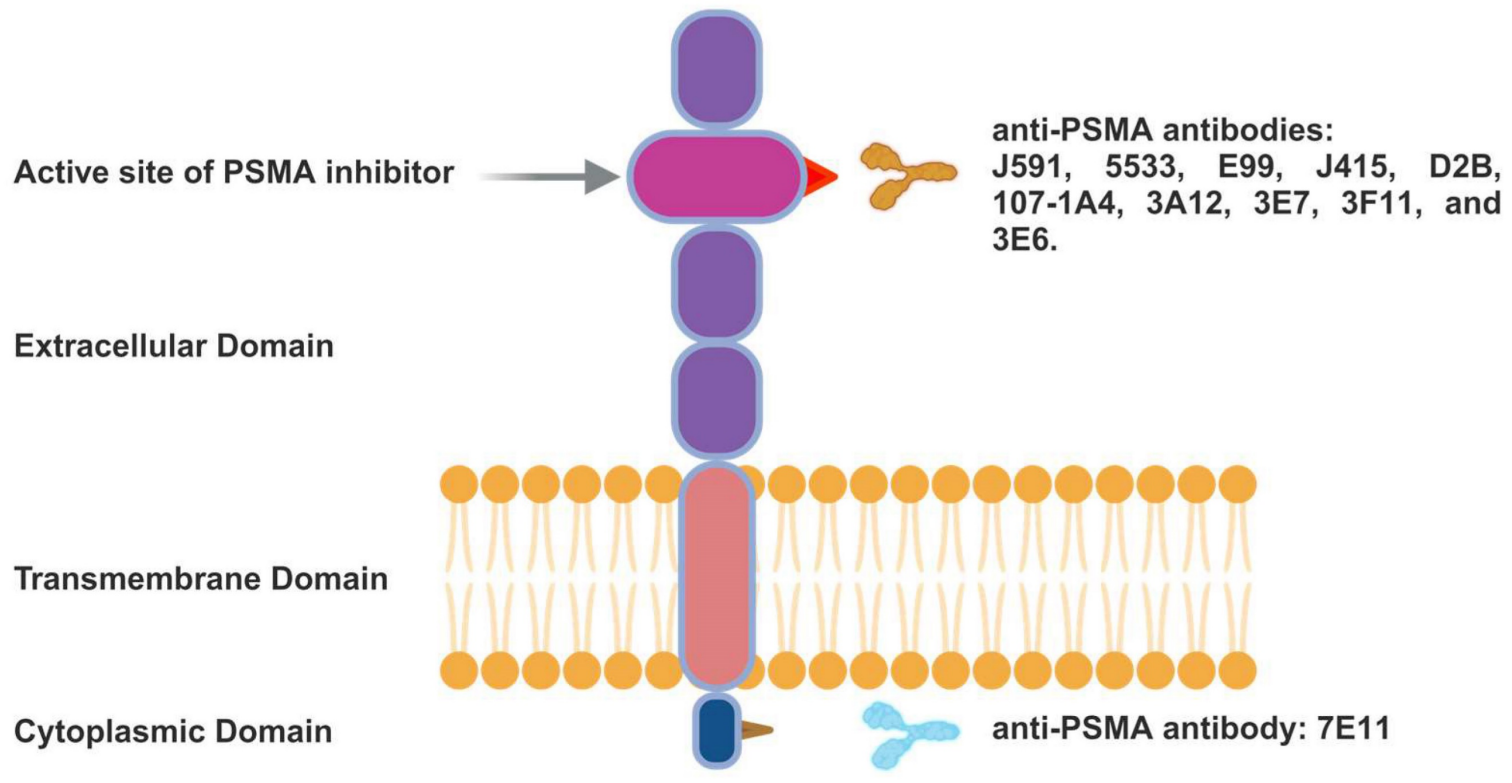
PSMA Glycoprotein Scheme. PSMA-specific antibodies and their recognised binding locations either in the N-terminal region, which is intracellular, or in the extracellular region.

mAbs were the initial clinically tested PSMA ligands. Based on whether their epitopes are in the intracellular or extracellular domains, anti-PSMA mAbs can be categorised. The structure of the PSMA glycoprotein and the recognised binding locations for PSMA-specific antibodies, including the *N*-terminal and extracellular regions, are illustrated in **Figure 4**. These mAbs can be radiolabelled or coupled with other agents to generate cytotoxic anticancer effects. The mAb 7E11 was the first radiolabelled antibody (^111^In-labelled capromab pendetide), which became approved by the FDA for PCa imaging (ProstaScint^®^, Cytogen) 29.

Because the mAb 7E11 binds only to the intracellular area of PSMA, the therapeutic efficacy of ProstaScint^®^ was restricted after receiving approval. mAbs targeting intracellular domain epitopes often react to necrotic or apoptotic cells only since their very hydrophilic nature prevents them from passing through the lipid membranes of living cells, and their bulky size (≈150 kDa) also serves as a major factor impeding their intracellular access 30.

Due to the limitations of the initial anti-PSMA mAbs, researchers have redirected their attention to the extracellular domain of PSMA. In 1997, the first four immunoglobulin G (IgG) mAbs that target the outer domain of PSMA were developed 31. Moreover, in this group of IgGs, mAbs are internalised by endocytosis 32. Their discovery prompted efforts to employ these PSMA mAbs to transport lethal cargos of drugs.

The first humanised mAb successfully used was the hu-J591, which provided the basis for radioligands and antibody-drug conjugates 33. In two independent phase I clinical trials, hu-J591, radiolabelled with either ^90^Y or ^177^Lu, was tested for the treatment of individuals with progressed CRPC 34. Both trials exhibited acceptable safety profiles, with thrombocytopenia and neutropenia of grade 3 signifying dose-limiting toxicities. The radiolabelled hu-J591 have shown anti-tumour properties, whereas in the ^177^Lu and ^90^Y studies, four of 35 and two of 29 candidates exhibited a significant decrease in prostate-specific antigen (PSA) of more than 50% over eight months, respectively. In contrast, 16 of 35 patients and six of 29 patients exhibited stable PSA levels for 60 days.

In comparison to [^90^Y]Y-hu-J591, [^177^Lu]Lu-hu-J591 is a minus-beta emitter with less energy and an extended half-life (2.7 vs. 6.7 days, respectively); additionally, it has a longer duration of tumour residence. Consequently, ^177^Lu had greater anti-tumour efficacy and caused less damage to healthy tissue 35. In addition, the emission of gamma rays by ^177^Lu renders it suitable for online therapy monitoring. These discrepancies prompted researchers to prioritise [^177^Lu]Lu-hu-J591 above its ^90^Y-labelled brethren. In a later phase II study, men with progressive mCRPC were treated with a single dose (65 or 70 mCi/m^2^) [^177^Lu]Lu-hu-J591, and disease response was evaluated after 12 weeks. Notably, 55% of the 47 patients admitted after illness progression and hormone therapy had previously received chemotherapy. After 12 weeks, 59.6% of the patients showed a decrease in PSA 36. Only 10.6% of individuals demonstrated a 50% or more drop in PSA levels. More patients in the 70-mCi/m^2^ dose group experienced a 30% or greater reduction in PSA than did those in the 65-mCi/m^2^ dose group (46.9% vs. 13.3%; P=.048). Survival was greater in the 70-mCi/m^2^ dose group than in the 65-mCi/m^2^ dose group (21.8 vs. 11.9 months; P=.03), although the hematologic toxicity grade 4 was greater. Only one out of the 12 patients with radiographically identifiable disease achieved a partial response. It was reported that 46.8% of the patients suffered reversible hematologic damage (grade 4 thrombocytopenia), and 25.5% experienced reversible neutropenia (grade 4 neutropenia) 37.

### Biomolecules vs. Low-molecular-weight Inhibitors and the Emergence of scFv, Nbs and Aptamers

**Figure 5:**
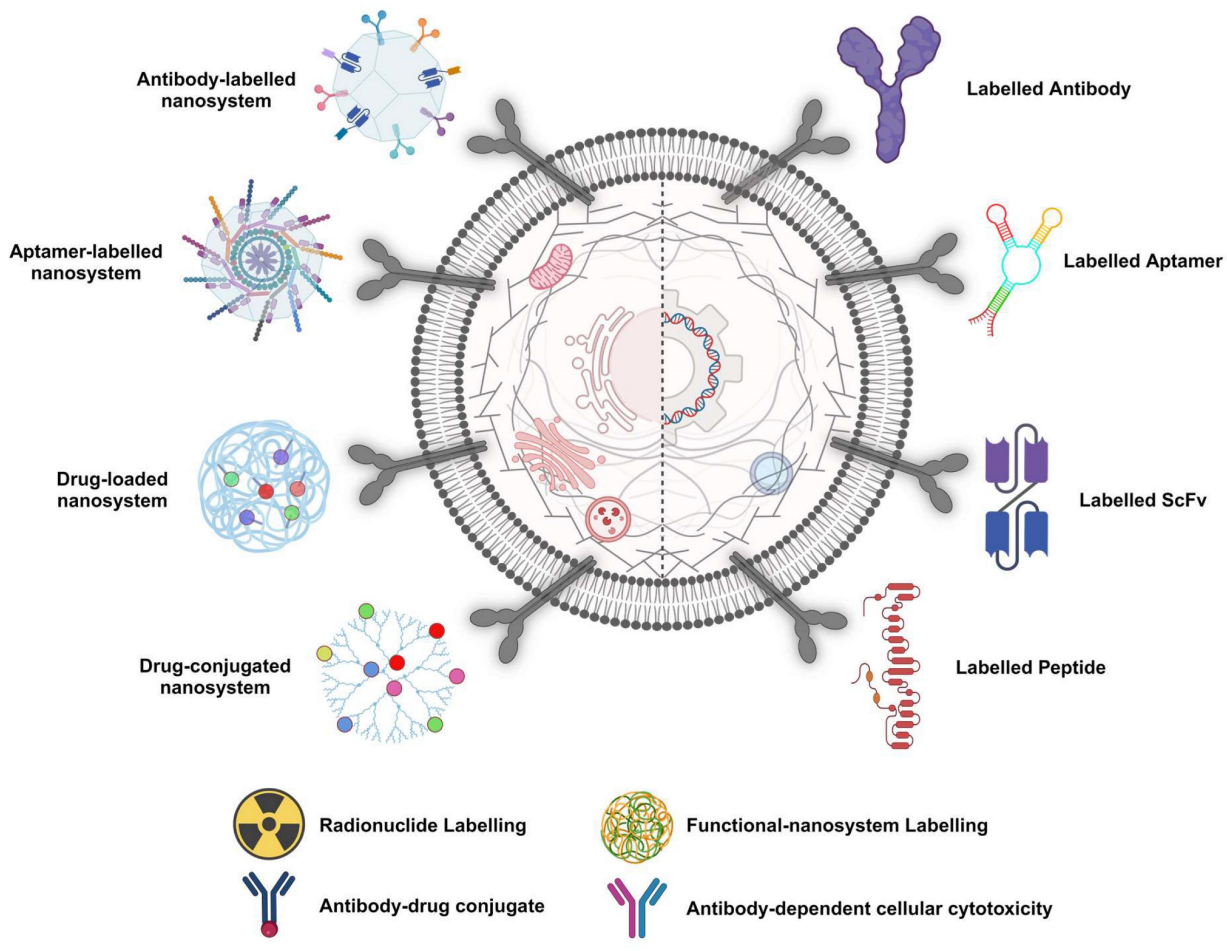
PCa diagnosis and treatment involve employing PSMA-specific ligand-targeting strategies. This entails the utilisation of various labelling candidates, including labelled antibodies. Notably, "siRNA" denotes short interfering RNA, and "scFv" represents a single-chain variable fragment.

Despite its high specificity for PCa and good safety profile, phase I and II investigations of [^177^Lu]Lu-hu-J591 have highlighted some significant drawbacks in using mAbs as the foundation of TRNT. One of those limitations is that mAbs have a protracted circulation time, which results in the higher exposure of non-targeted organs. Furthermore, compared with small molecules, mAbs do not penetrate solid tumours efficiently; consequently, PSMA compounds with a lower molecular weight were created (**Figure 5**). One approach to reduce molecular weight is by using diverse Ab regions and fragments. Ab fragments, including single-chain fragments (scFvs), are currently being studied for use in radiotheranostics. To create a scFv, portions of the variable heavy and light chains of mAbs are joined. The scFv domain, an IgG1 hinge, and a CH_3_ domain make up the minibodies 38.

In a phase I study, patients with metastatic PCa were applied with the zirconium-89 (^89^Zr)-labelled desferrioxamine-IAB2M minibody ([^89^Zr]Zr-DF-IAB2M), which demonstrated its efficacy and safety in targeting skeletal and lymph node metastases 39. Since imaging was conducted 48 hours post-injection (p.i.), the results of comprehensive clinical interpretation were inconclusive. In a later phase II study, [^89^Zr]Zr-DF-IAB2M was comparable in performance to gallium-68 (^68^Ga)-labelled PSMA-11 for PET/CT before prostatectomy ([^68^Ga]Ga-PSMA-11). In preclinical *in vivo* trials, favourable findings were achieved using smaller scFv radioligands. An scFv derived from a D2B antibody and labelled with ^124^I revealed improved cellular uptake efficiency and increased specificity in PSMA-positive cells at an appropriate period post-infusion 40.

Nanobodies are antigen-binding heavy chain-only Abs that come from the Camelidae family and are thus known as heavy chain-only Abs (VHHs) 41. The smallest functional Ab derivatives combine high affinity with increased diffusion in tumour tissues, improved pharmacokinetics, and decreased immunogenicity 42. One of the distinguishing features of nanobodies is their capacity to target antigenic epitopes in areas that are difficult for large molecules, such as traditional mAbs, to reach 28. To date, PSMA-targeted nanobodies have been produced primarily for PCa imaging and evaluated *in vitro* and in xenograft-bearing mice 43. The ^111^In-DTPA-labelled engineered expression-modified nanobody JVZ-007, with a myc tag and a cys tag ([^111^In]In-JVZ007-c-myc-his and [^111^In]In-JVZ007-cys), was presented by Chatalic *et al*. Such nanobodies can target PSMA-positive tumours and be cleared rapidly from the blood. For the first time, in 2017, the therapeutic use of the PSMA nanobody was examined 44; this study revealed that the JVZ-008 nanobody labelled with bismuth-213 (^213^Bi, [^213^Bi]Bi-JVZ-008) can target PCa quickly and effectively in mice with PSMA-positive LNCaP xenografts. The VHH nanobody used to target PSMA was developed by Zare and colleagues, and it showed outstanding *in vitro* specificity and affinity for LNCaP cells. Furthermore, the PSMA nanobodies PSMA6 and PSMA30 labelled with ^99m^Tc and ^111^In for SPECT imaging revealed powerful tumour penetration and rapid clearance in PSMA-expressing xenografted animals 43.

In addressing the challenges, PSMA-specific RNA aptamers offer a promising avenue for enhancing drug delivery, given their target cell specificity and reduced immunogenic responses. With their ease of development and potential for precise targeting, aptamers have emerged as a key focus for improving PCa radiotheranostics 45. However, during the exploration of ligand-conjugated nanocarriers, their tumour accumulation is notably low, <0.01%, highlighting the need for more effective delivery systems. 46. Traditional chemotherapy, while effective at inhibiting PCa proliferation at later stages, is associated with off-target effects and critical side effects due to its lack of specificity. Bioactives carried by targeted nanoparticles (NPs) constitute a potent strategy for enhancing the precision and sensitivity of PCa diagnosis and treatment. Aptamers, with their high specificity and ability to bind to PCa-linked cell membrane protein markers, are pivotal in designing modified NPs for site-specific delivery 46. This approach not only promises to improve the management of PCa by ensuring the targeted delivery of therapeutic agents but also minimises the toxic effects associated with conventional chemotherapy. Through sophisticated extraction processes from nucleic acid libraries, these synthetic ligands are adept at targeting a broad spectrum of molecules. When combined with nanomaterials such as quantum dots (QDs), they can form potent bioconjugates for advanced aptasensing applications. These conjugates have shown remarkable efficacy in detecting a variety of cancers and their biomarkers, including prostate-specific antigens and nucleolin, thereby enhancing the precision of cancer diagnostics 47.

These reagents have been advanced in multiple laboratories in recent years by truncation, extension, and modification. Further alteration of 2'-purine subunits has the potential to yield further improvements. Additional alterations or sequence replacements may enhance an aptamer's folding, stability, or conjugation capability. Considering that only 6.00E-08% of the potential 1024 unique aptamers were applied to the original *in vitro* xPSM, new aptamer selection procedures or libraries may also uncover novel PSMA-targeting aptamers with superior size or affinity. New RNA synthesis or nucleotide modification methods may reduce the cost of aptamer synthesis or simplify Good Manufacturing Praxis (GMP), advancing the clinical application or translation of these materials 48. In PSMA aptamer research, there are still lingering questions; for instance, structural investigations of aptamer folding and docking have not been performed. For crystallisation, crystallography demands highly pure aptamers and proteins with homogeneous RNA folding and three-dimensional structures. Unfortunately, it has been challenging to create these crystals, presumably due to the heterogeneous folding of the A10-3 aptamer. However, the mechanism of action of aptamer-siRNA chimaera-AsiC, endosomal escape, and processing for effective RNA have not been fully elucidated 49.

After all, it is essential to recognise that PSMA aptamers are only a tiny part of the overall endeavour to create PSMA-targeted diagnostic and therapeutic agents 50 51. To maximise the potential effectiveness of these agents for PCa-afflicted men, it will be essential to understand the advantages and disadvantages of each of these PSMA-targeting tools.

## PSMA-targeted Radiotheranostics: Inhibitors

The need to use lower-molecular-weight targeting agents while maintaining PSMA specificity led to the development of various PSMA inhibitors. Motor neuron (MN) death has been linked to glutamate excitotoxicity in amyotrophic lateral sclerosis (ALS) and familial ALS (FALS). A neuroprotective strategy including potent and selective inhibitors of GCPII, which converts the abundant neuropeptide NAAG to glutamate, could protect MNs in *in vitro* and animal models of FALS. Numerous studies indicate that GCPII inhibitors decrease MN cell death in each of these circumstances by decreasing glutamate concentrations. Selective GCPII inhibitors are becoming a significant area of GCPII research due to the neuroprotective effect obtained from reducing GCPII enzyme activity in the brain. Additionally, for GCPII-based imaging of PCa, inhibitors can be employed as "homing devices" 22. Over the past two decades, several GCPII inhibitors with various chemical scaffolds, almost all of which originated from NAAG, have been developed.

In 1996, the neuropeptidase inhibitor NAALADase was initially developed to study and treat problems in the nervous system 52. The phosphonate derivative 2-phosphonomethyl pentanedioic acid (2-PMPA) serves as a substrate or analogue for the transition state. Since NAALADase and FOLH1 both have the same enzymatic function, research has shifted to identifying and using 2-PMPA as a low-molecular-weight GCPII inhibitor. Phase I trial evaluating [^18^F]2-PMPA (BAY 1075553) for PET/CT imaging found this analogue less effective than [^18^F]FET because it had low selectivity for lymph node and bone marrow metastases 53.

### Phosphorus-based GCPII Inhibitors

**Figure 6:**
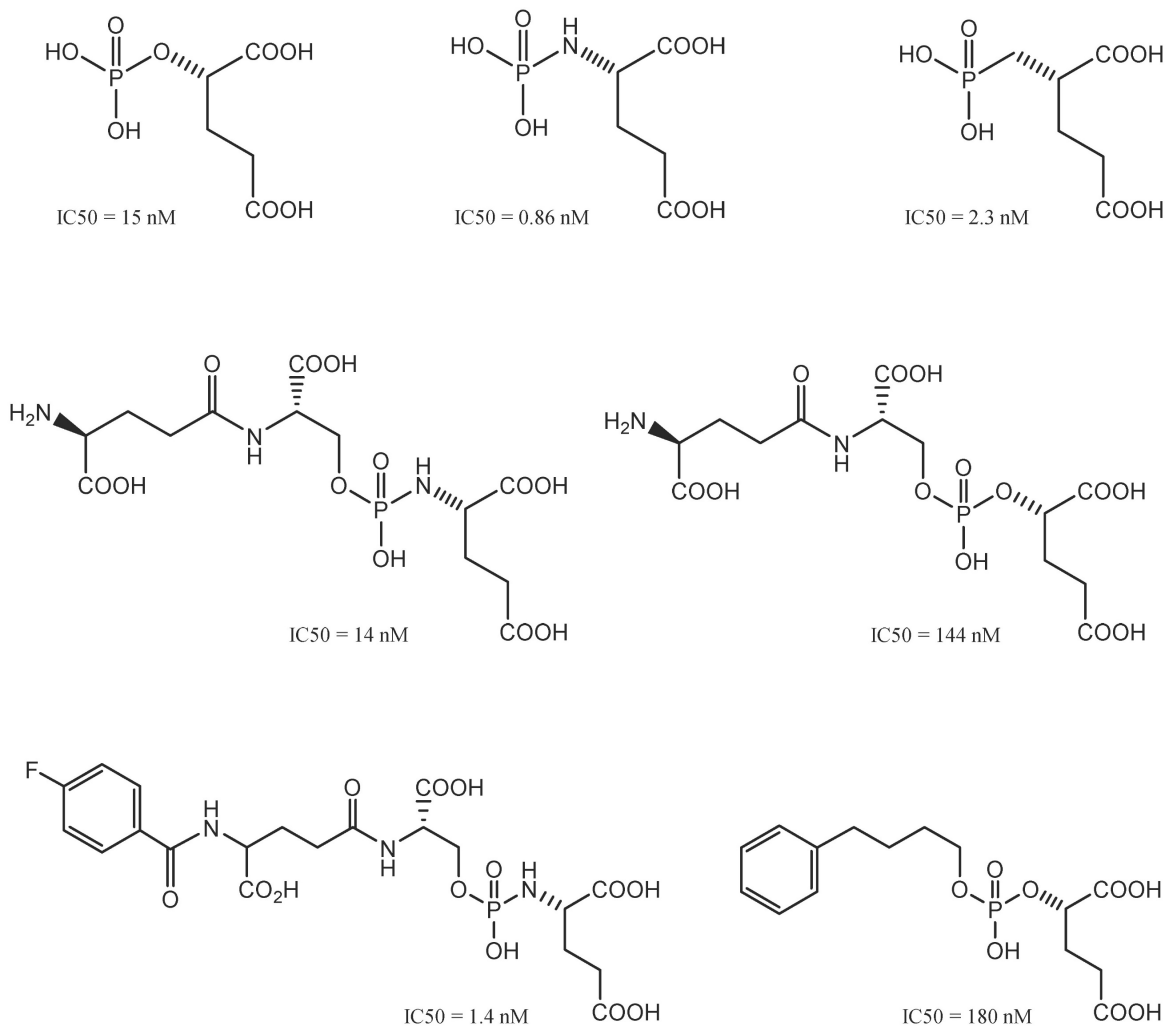
Phosphorus-based GCPII inhibitors. In this class, a variety of phosphonate-, phosphinate-, and phosphoramidate-based PSMA-targeting compounds were developed. Phosphoramidate inhibitors represented the most promising pharmacophores so far. Reproduced with permission from Springer Nature publisher 195.

Initially, GCPII inhibitors were described as phosphorus-containing inhibitors; they were essential for comprehending the mechanism through which GCPII operates in the body 54, in which the tetrahedral phosphorus moiety is similar to the transition state of peptide bonds (tetrahedron) (**Figure 6**).

Then, when phosphinic and phosphoramidate scaffolds (such as NAALADase transition states) were found, the race to develop the best low-molecular-weight PSMA inhibitors began 55. The phosphoramidate compound [^18^F]CTT1057 showed potential for PSMA-based radiodiagnostics in a recent phase I study 56. Specifically, the study delineated an average total effective dose of 0.023 mSv/MBq, indicating favourable dosimetric characteristics 56. Notably, the kidneys, as the primary organ of concern for radiopharmaceutical accumulation, exhibited the highest absorbed dose at 0.067 mGy/MBq, while the salivary glands recorded an absorbed dose of 0.015 mGy/MBq. Furthermore, the diagnostic ability of [^18^F]CTT1057 was evident in its ability to detect 97 metastatic lesions in a cohort of 15 patients, demonstrating its utility in identifying disseminated PCa with high sensitivity 56. In the detection of bone metastases, [^18^F]CTT1057 proved effective by identifying 44 out of 56 bone metastases (78.5%), a finding that was comparably corroborated by bone scans. Additionally, [^18^F]CTT1057 demonstrated an ability to detect lymph nodes, identifying eight out of 32 lymph nodes (25%) that were not previously enlarged according to conventional CT size criteria. These quantitative outcomes not only reinforce the potential of [^18^F]CTT1057 but also highlight its significant role in advancing diagnostic accuracy 56. Nonetheless, the biological instability and unfavourable toxicity profiles of phosphinic- and phosphoramidate-based compounds have hindered their clinical development. On the other hand, urea-based inhibitors are generally easier to synthesise and modify, which makes their use more favourable despite having a similar molecular makeup 57.

### Urea-based GCPII Inhibitors

**Figure 7:**
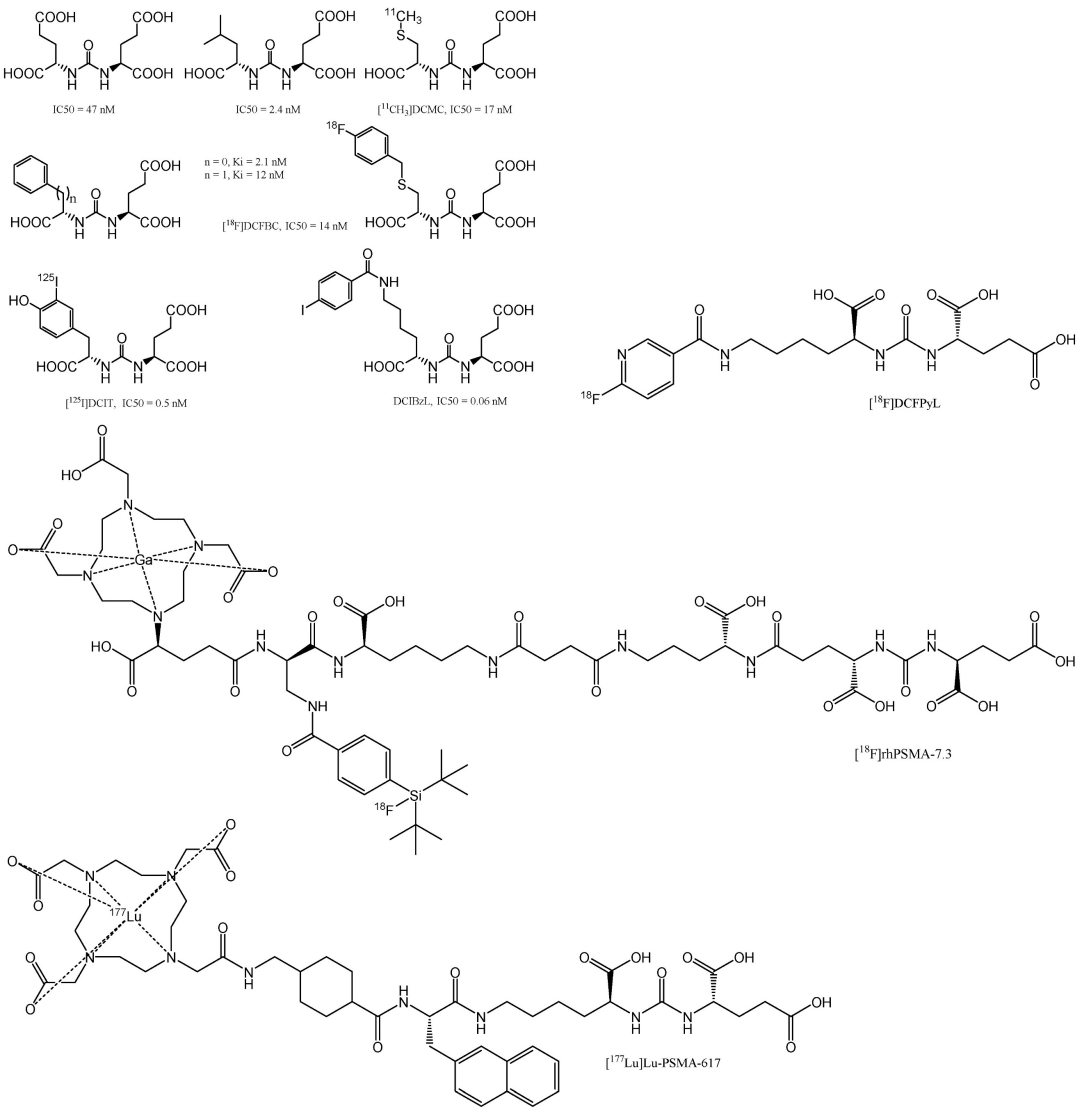
Urea-based GCPII inhibitors. This group of ligands delivered most of clinically relevant PSMA-targeting compounds. The figure also depicts exemplary FDA-approved radioligands [^18^F]DCFPyL (PYLARIFY^®^, Lantheus Holdings), [^18^F]rhPSMA-7.3 (POSLUMA^®^, Blue Earth Diagnostics) and [^177^Lu]Lu-PSMA-617(Pluvicto^®^, Novartis). Reproduced with permission from Springer Nature publisher 195.

Urea-based agents represent the most popular class of selective GCPII inhibitors discovered in the 21^st^ century 58. Urea-based PSMA inhibitors (**Figure 7**) consist of two amino acids joined by a urea group in their backbone (glutamate-urea-X, where X refers to lysine, cysteine, or another glutamate). Most inhibitors require a glutamate residue to attach to the S1′ pocket of the enzyme, where the planar peptide bond of the sliced substrate is subsequently imitated by the ureido group 59. Therefore, more urea-based inhibitors, in diverse ways, fluorophores, toxins, and radionuclides, are interconnected and have been developed and successfully utilised for the diagnosis and treatment of PCa 60. DCIBzL is one of the most effective GCPII inhibitors; it features a phenyl ring that binds to the hydrophobic pocket at the S1 site and is an outstanding example of this type of molecule 61.

^123^I-MIP-1095 and ^123^I-MIP-1072 were the first radiolabelled urea-based inhibitors investigated in humans, where the glutamate-urea-lysine motif was used in both drugs 62. In December 2020, the FDA approved PSMA inhibitor [^68^Ga]Ga-PSMA-11 (Locametz^®^, Novartis/ ILLUCCIX^®^, Telix Pharmaceuticals) for PCa imaging 63. Using a similar approach, Chen and co-workers attached [^18^F]fluoropyridyl to a Glu-urea-Lys backbone. In May 2021, after publishing phase 2/3 clinical trials (OSPREY, NCT02981368 and CONDOR, NCT03739684), the FDA also approved this PSMA inhibitor, known as [^18^F]DCFPyL (PYLARIFY^®^, Lantheus Holdings) for PCa imaging 64. Notably, [^18^F]DCFPyL PET/CT has emerged as an overly sensitive diagnostic tool for detecting lesions following primary definitive therapy, as evidenced by its performance in a phase II/III OSPREY study cohort, where it achieved a detection sensitivity of 95.8% for extra-prostatic lesions in patients with radiological signs of recurrence 65. The imaging agent is noted for its high tumour uptake, which is comparable to that of [^68^Ga]Ga-PSMA-11 and shows improvement over [^18^F]DCFBC. Additionally, [^18^F]DCFPyL exhibits favourable clearance and normal tissue distribution, ensuring that radiation doses adhere to the FDA guidelines 65. However, the interpretation of these findings is limited by the relatively low frequency of histopathological confirmation of the detected lesions, which is a crucial aspect for validating the diagnostic accuracy of such imaging agents. This limitation highlights the need for further studies incorporating histopathological standards to fully ascertain the clinical utility of [^18^F]DCFPyL in the management of PCa 66.

### Thiol-based GCPII Inhibitors

**Figure 8:**
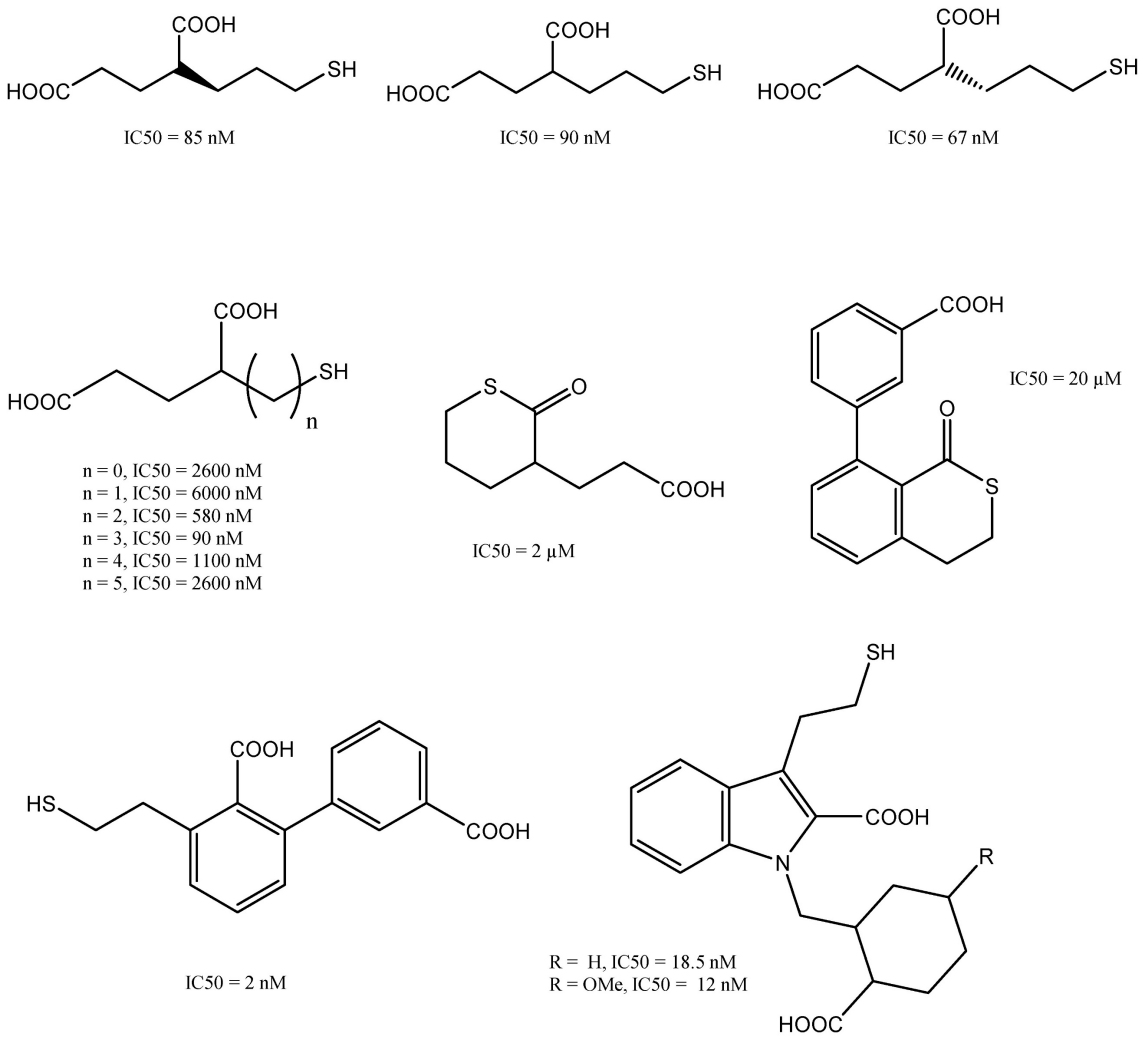
Thiol-based and other GCPII inhibitors. These PSMA-targeting compounds were mainly developed as analogous to phosphorus-based GCPII inhibitors. However, this class of ligands demonstrated overall low stability due to high sensitivity to oxidation. Reproduced with permission from Springer Nature publisher 195.

The thiol-based inhibitor 2-(3-mercaptopropyl) pentanedioic acid (2-MPPA) was the first GCPII inhibitor to be administered orally 67. PSMA ligands that contain thiol groups (**Figure 8**) tend to form disulphide bonds, which can result in reduced metabolic stability that limits their clinical usefulness 68. Investigations into further compounds with a zinc-binding hydroxamate group revealed that the inhibitory effect of GCPII was inferior to that of compounds based on phosphonate or thiol groups 69.

### Hybrid GCPII Inhibitors

In October 2020, Tolvanen *et al*. conducted a pioneering first-in-human study that explored the safety, biodistribution, and radiation dosimetry associated with a novel ^18^F-labelled urea-based radiohybrid PSMA ligand designated as [^18^F]rhPSMA-7.3 70. In a phase I open-label study, the uptake kinetics of [^18^F]rhPSMA-7.3 were evaluated 71. Another study explored the utility of [^18^F]-rhPSMA-7.3 for pre-operative efficacy for N staging in patients with unfavourable intermediate- to very high-risk profiles, as validated by histopathology. This research especially provided insights into primary PCa staging 72. In May 2023, a significant milestone was achieved with [^18^F]rhPSMA-7.3 FDA's approval (Posluma^®^, Blue Earth Diagnostics) for the PET assessment of PSMA-positive lesions in patients with PCa who were receiving initial definitive therapy or who were experiencing suspected recurrence, as evidenced by elevated serum PSA levels. In the phase III trials LIGHTHOUSE (NCT04186819) and SPOTLIGHT (NCT04186845), the [^18^F]rhPSMA-7.3 injection demonstrated the ability to detect distant metastatic lesions and provided a clinically meaningful correct detection rate, increasing upstaging of disease in recurrent PCa 73.

This radiohybrid concept could be described as follow. First, both the SiFA and the chelator can be labelled in an independent manner using the unprotected precursor, resulting in either [^18^F]M-rhPSMA (M = metal) or [^19^F]R-rhPSMA (R = radiometal), the latter of which can be used for imaging (e.g., ^68^Ga for PET, ^111^In for SPECT), or TRNT (e.g., ^177^Lu). The corresponding radiopharmaceuticals, for example, [^18^F]^69/71^Ga-rhPSMA and [^19^F]^68^Ga-rhPSMA, are chemically identical molecules. Thus, they represent monozygotic chemical twins that should result in almost identical PET scans, with only slight differences determined by the nuclear properties of the chosen radioisotope. In addition, when ^18^F is combined with a therapeutic radioisotope, such as ^177^Lu, the resulting twins, [^18^F]^175/176^Lu-rhPSMA or [^18^F]^177^Lu-rhPSMA, could, for the first time, truly bridge PET imaging and TRNT. Although speculative, such tracers might be interesting tools for pre-therapeutic patient stratification, pre-therapeutic dosimetry, and TRNT with a single tracer 74. In one instance, the radioligand [^177^Lu]Lu-rhPSMA-7.3 was evaluated in a pre-therapeutic dosimetry study involving PCa patients 75. Compared to [^177^Lu]Lu-PSMA I&T, the application of [^177^Lu]Lu-rhPSMA-7.3 resulted in a significantly greater tumour dose, albeit with greater kidney accumulation. Another study compared the four isomers of [^177^Lu]Lu-rhPSMA-7 ([^177^Lu]Lu-rhPSMA-7.1, -7.2, -7.3, and -7.4), along with the novel radiohybrid ligands [^177^Lu]Lu-rhPSMA-10.1 and -10.2, which were compared to the state-of-the-art compounds [^177^Lu]Lu-PSMA I&T and [^177^Lu]Lu-PSMA-617. The comparative evaluation included affinity studies (IC_50_), internalisation experiments, and lipophilicity measurements 74. [^177^Lu]Lu-rhPSMA-10.1 has shown promising results in preclinical assessments 76. However, further clinical studies are required to validate these promising preclinical results 74. Notably, the efficacy of [^18^F]rhPSMA-7.3 in PET imaging has been subjected to various analyses, including a comparison of detection sensitivities on a right vs. left basis, where it demonstrated a sensitivity of 61.5% 77. Additionally, its sensitivity for identifying pelvic nodal metastases was 66.7%, according to another study 72. [^18^F]rhPSMA-7.3 has been recognised for its tolerability and high detection rate, achieving an overall detection rate of 83% among patients with biochemically recurrent PCa 66. Despite these strengths, the utility of [^18^F]rhPSMA-7.3 is tempered by limitations in the data, particularly the low frequency of histopathologically validated lesions. This gap underscores the need for further research incorporating histopathological standards to fully evaluate the diagnostic accuracy and clinical relevance of [^18^F]rhPSMA-7.3 in PCa management 66.

## Linkers and Chelators

The off-target uptake of PSMA-targeted radioligands is complex and can be influenced by several factors. While chelators and targeted pharmacophores play a role, other factors, such as the biological properties of the tissues and the specific characteristics of the radioligands, including complex stability, can also contribute 78. There is indeed ongoing debate about whether the kidneys and salivary glands uptake of PSMA-targeted radioligands is mediated by PSMA. Some studies suggest that the high and sustained off-target uptake of PSMA-targeted radioligands in normal organs reduces their sensitivity for detecting lesions in and adjacent to those organs 78. Another study indicated that the uptake of PSMA-targeted radioligands in the kidneys and salivary glands can be substantially reduced without significantly impacting tumour uptake by adding cold PSMA inhibitor PSMA-11 79. In addition, studies have suggested that the degree of PSMA expression and the fraction of PSMA positive cells correlate with the uptake of PSMA-targeted radioligands and thus their efficacy 78. While there is evidence suggesting both PSMA-mediated uptake and the role of chelators or pharmacophores, the exact underlying mechanisms are still under debate. However, further research is needed to fully understand these mechanisms and develop strategies to reduce off-target effects.

Chelation typically requires harsh conditions, which limits its suitability for tagging biological vectors. The ideal chelator would allow labelling under favourable conditions (near-neutral pH and low to moderate temperatures [37-42°C]) and be thermodynamically and kinetically stable. Numerous new chelators with improved characteristics have been developed, making them potential candidates for future therapeutic applications 80.

The focus of low-molecular-weight PSMA ligands has recently shifted from chelator to linker area modifications. Additionally, there is growing evidence that the PSMA-binding entity and overall structure, including the chelator and linker moieties, affect the binding affinity and internalisation ratios. Notably, the pharmacokinetics, pharmacodynamics, and bioavailability of PSMA-targeting low-molecular-weight inhibitors are significantly impacted by changes in the linker and chelator sites. Short linkers and non-polar moieties that aim to open the PSMA-binding funnel can be used to increase the affinity of PSMA for binding, as demonstrated by Bařinka *et al*. 81. Further investigations revealed that in the PSMA catalytic sub-pocket, powerful PSMA inhibitors interact with Zn^2+^ ions, critical amino acids, and lipophilic and cationic interactions in the S1 lipophilic region. Additionally, Zhang *et al*. discovered a second arene-binding region that can engage in aromatic stacking interactions with low-molecular-weight inhibitors 82.

Radionuclides were initially introduced *via* straightforward nucleophilic substitution of aromatic ring systems linked to urea-based binding moieties 83. The clinically-relevant examples are addressed to radioiodinated MIP-1072 and MIP-1095, according to a study performed by Barrett and his co-workers 62. Pre-clinical imaging studies were used to compare the therapeutic efficacy of these various medications, and it was projected that adding a second urea group to MIP-1095 would boost its lipophilicity and make it more potent than adding an amine group to MIP-1072 84. Even though systemic drugs are commonly cleared rapidly from the blood, the renal clearance of [^131^I]MIP-1072 was significantly faster, presumably due to structural conformational arrangements 62; hence, [^131^I]MIP-1095 was chosen for further clinical testing 85.

By synthesising para-substituted benzoic acid and small compounds based on the EuK binding motif, Kiess and his group utilised findings from diagnostic tests to develop the first astatine-bearing low-molecular-weight inhibitor, astatine-211-labelled DCAtBzL 83. The equivalent absorption and chemical similarity between iodine- and astatine suggested the use of iodine compounds as surrogates for astatine in preclinical settings. Nonetheless, the potency of these compounds was severely compromised by the high renal absorption. To remedy this problem, Childers *et al*. examined the constitutional isomers of these inhibitors. While keeping the structural analogues of the Glu-urea-Glu binding entity (i.e., the linkers' sizes and their functional sub-units), they enhanced the tumour-to-kidney ratio in mice by eight-fold 21 hours p.i. Vaidyanathan *et al*. reported that adding a guanidino group to the aromatic ring of an inhibitor massively altered its biodistribution and pharmacokinetics 86. Moreover, changes in quinolone derivatives appear to be useful for diagnostic tracers and might be utilised as models for prospective cancer treatments 87.

**Figure 9:**
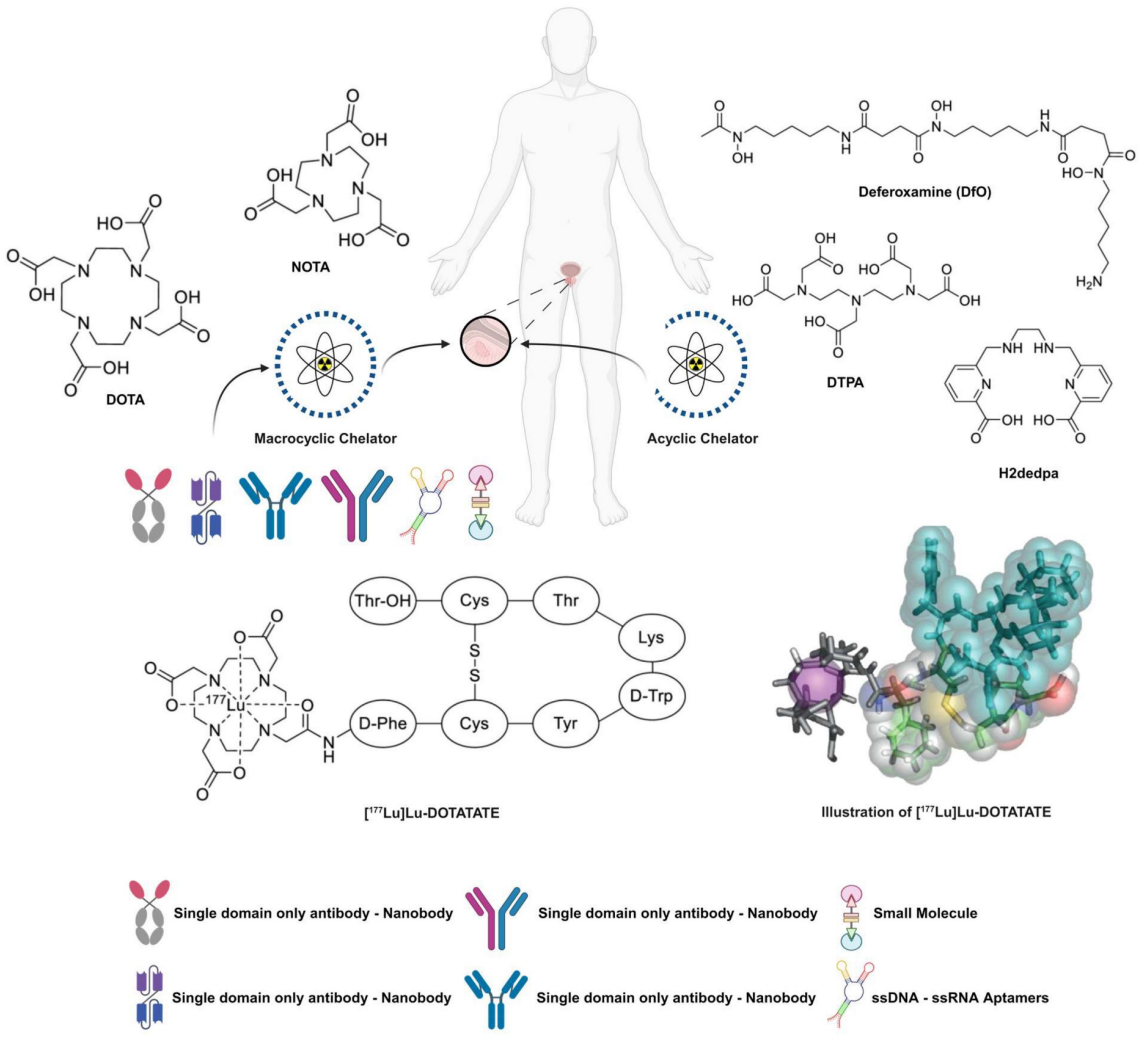
Examples of typical macrocyclic and acyclic chelators. Includes a comprehensive array of ligands, with an example of the commonly used SSTR2-targeting [^177^Lu]Lu-DOTA-TATE (Lutathera^®^). Reproduced with permission from Elsevier publisher 80.

Unlike radionuclides such as ^131^I or ^211^At, which have already been discussed, the inclusion of radiometals in PSMA-targeting inhibitors requires an acyclic, macrocyclic or hybrid complexing agent (chelator). The most common macrocyclic chelator used in therapy is DOTA, which serve as complexing agents for ^177^Lu^3+^ and other trivalent cations of great therapeutic importance (**Figure 9**). In 2010, Banerjee *et al*. published the first DOTA-based inhibitor that targets PSMA and has a EuK-binding entity 88. The examined compounds shared the suberic acid and L-lysine structural components of the linker region while maintaining the primary linker components. By altering the DOTA chelator through the addition of two L-phenylalanine units, the target-to-tissue ratios improved, and the tumour uptake was comparable to that of the parent structure. In subsequent investigations, further changes to the linker and chelator areas resulted in even more significant gains 60. Exploration of the modification of the DOTA chelator, in addition to adjustments in the linker and chelator domains, has yielded insightful quantitative data that elucidates the impact of these alterations on the pharmacokinetics and stability of radiopharmaceutical compounds 89. Notably, the strategic incorporation of two L-phenylalanine units adjacent to the DOTA chelator while maintaining the integrity of the main linker elements resulted in enhanced target-to-tissue ratios 90. This modification achieved tumour uptakes that were on par with the original structure, suggesting an optimisation in the balance between specificity and systemic distribution.

Further investigative efforts focused on the development of acyclic DFO*-NCS ester and DFO- squaramide ester, novel conjugation analogues of the traditional DFO chelator, which have demonstrated more stable ^89^Zr complexes 91. Comparative studies highlighted that the trastuzumab conjugated with both [^89^Zr]Zr-DFO*-NCS and [^89^Zr]Zr-DFO*Sq exhibited remarkable *in vitro* stability, outperforming their [^89^Zr]Zr-DFO counterparts across all tested conditions 91. This superior stability was notably preserved even 30 days p.i. equivalent to approximately nine half-lives of ^89^Zr. At this juncture, despite residual activity ranging from 20 to 40 kBq in animal models, the imaging quality has remained high enough to delineate activity in critical organs such as the liver, kidneys, and joints of both the upper and lower limbs 92. These findings collectively underscore the potential of strategic modifications to the chelator and its associated linker areas for enhancing the pharmacological profile of radiolabelled compounds. By achieving significant gains in stability and tissue targeting, these advancements represent pivotal steps forward in the optimisation of radiotracers and radiopharmaceuticals 90, 91.

Another example could be demonstrated by the linkage of the binding motif to either HBED-CC (diagnostic) or DOTA (theranostic) as chelators while maintaining the EuK unit using a linker made of 6-aminohexanoic acid. At room temperature, HBED-CC, an acyclic radiometal chelating agent, can label ^68^Ga in 5 min with 98% radiochemical yield and 99% radiochemical purity after isolation. This method provides distinctive preclinical information as well as significant facets of the [^68^Ga]Ga-PSMA-11 production method 93. The DOTA derivative responded similarly to the other inhibitors; however, the HBED-CC-based compound (PSMA-11) significantly increased cell internalisation. The study of PSMA theranostics has also greatly benefited from further research into linker modifications of low-molecular-weight urea-based inhibitors 94.

In 2015, preclinical data on PSMA I&T and PSMA-617 were disclosed. Both radiopharmaceuticals were proposed for TRNT 95 based on their distinct pharmacokinetic profiles. PSMA I&T and PSMA-617 are based on the urea motif; the chelator in both compounds allow them to host trivalent radionuclides. Weineisen *et al*. showed that adding DOTAGA as a chelator to PSMA I&T would be beneficial 96. To elaborate further, this research has highlighted the significant advantages of DOTAGA over traditional DOTA in terms of tumour-targeting efficiency. Initially, the study's findings pointed to a pronounced improvement in tumour absorption by DOTAGA-variant compared to DOTA-variant, though no detailed numerical data were provided to quantify this enhancement. It was also noted that the performance of [^68^Ga]Ga-PSMA I&T matched that of [^68^Ga]Ga-PSMA-11, suggesting its competitiveness 97.

The study detailed quantitative radiochemical yields for both ^68^Ga and ^177^Lu labelling under optimised conditions, which speaks volumes about the efficiency of DOTAGA in radiopharmaceutical preparations. For ^68^Ga labelling, the conditions were set at 3 nmol, with a solution concentration of 5.0M NaCl and 2.7M HEPES (approximately a 5:1 ratio) at a pH range of 3.5 to 4.5 for 5 minutes at 95°C. For ^177^Lu labelling, the procedure required 0.7 nmol in 0.1M NH_4_OAc, with a pH of 5.5, for 30 minutes at 95°C. The specific activities achieved were fairly high, with ^68^Ga-labelled analogues reaching 250 to 300 GBq/μmol and ^177^Lu complexes at 38 GBq/μmol. Furthermore, compared with traditional DOTA ligands, DOTAGA derivatives exhibited greater hydrophilicity, with log P values of -3.6 ± 0.1 for ^68^Ga and -3.9 ± 0.1 for ^177^Lu, suggesting that an improved physicochemical profile could enhance tumour targeting and biodistribution 97. Additionally, these derivatives also achieved an approximately two-fold increase in the specific internalisation of both ^68^Ga- and ^177^Lu-labelled DOTAGA analogues compared to that of their DOTA counterparts. This enhanced cellular uptake is favourable for the efficacy of TRNT and diagnostic imaging 98. Rapid proteolytic cleavage of the radiolabelled inhibitor was achieved by switching out the L-amino acids to afford D-amino acid analogues, which also improved the pharmacokinetic profile and stability *in vivo*. The peptidomimetic linker unit was created by replacing D-phenylalanine with 3-iodo-D-tyrosine to improve the lipophilic interaction of the peptide with the distant arene-binding site in the PSMA-binding pocket 98 99. Biodistribution studies in LNCaP tumour-bearing CD-1 nu/nu mice complemented these findings, offering a detailed view of the *in vivo* behaviour and its potential for clinical application 99. Initial patient studies with [^68^Ga]Ga-PSMA I&T have demonstrated significant tumour uptake, with tumour-to-background ratios reported at 29.6±13.5 for the SUV mean ratio and 33.5±9.7 for the SUV max ratio 99. These metrics not only affirm the high-contrast imaging capabilities of [^68^Ga]Ga-PSMA I&T but also underscore its specificity and efficacy in identifying PSMA-expressing PCa lesions.

PSMA-617 was developed using a method that involved tailor-made alterations to the linker region of DOTA-conjugated inhibitors 95. Due to the remarkable reduction in tumour-targeting properties that were observed when HBED-CC was replaced with DOTA in PSMA-11, linker modifications were performed to enhance the interaction of the inhibitor with the PSMA binding pocket 100. The original set of compounds had several aromatic rings and configurations in the linker region. This shows how vital the aromatic moieties are between the EuK entity and DOTA. The highest affinity for PSMA was observed for a compound containing three aromatic rings in the linker region, although this molecule had lower internalization rate. It was found that at least one aromatic moiety with a rigid shape in the linker region seem to be favorable. For instance, PSMA-617, a compound with a linker consisting of 2-naphthyl-L-alanine (2-Nal) and *trans*-4-(aminomethyl) cyclohexane carboxylic acid (AMCH), had the best performance. Modifications showed that 2-Nal's chirality and its constitutional isomerism affected the drug's properties significantly. Benešová *et al*. found a series of inhibitors, with the only other likely structural change being a phenyl group substituted for the cyclohexyl ring; the kidney clearance was slower due to its greater lipophilicity, even though this alteration was likely to be attractive 100.

**Figure 10:**
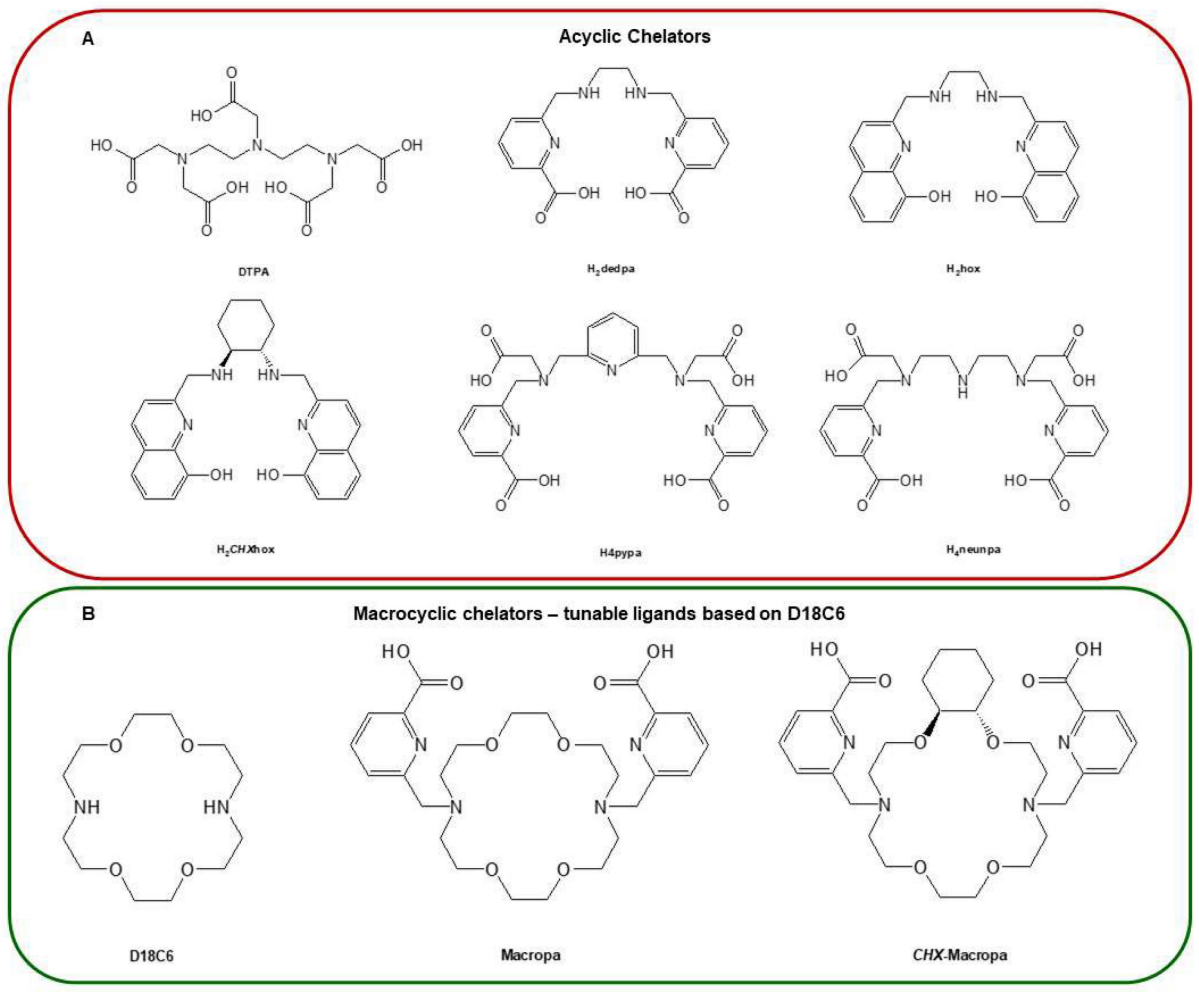

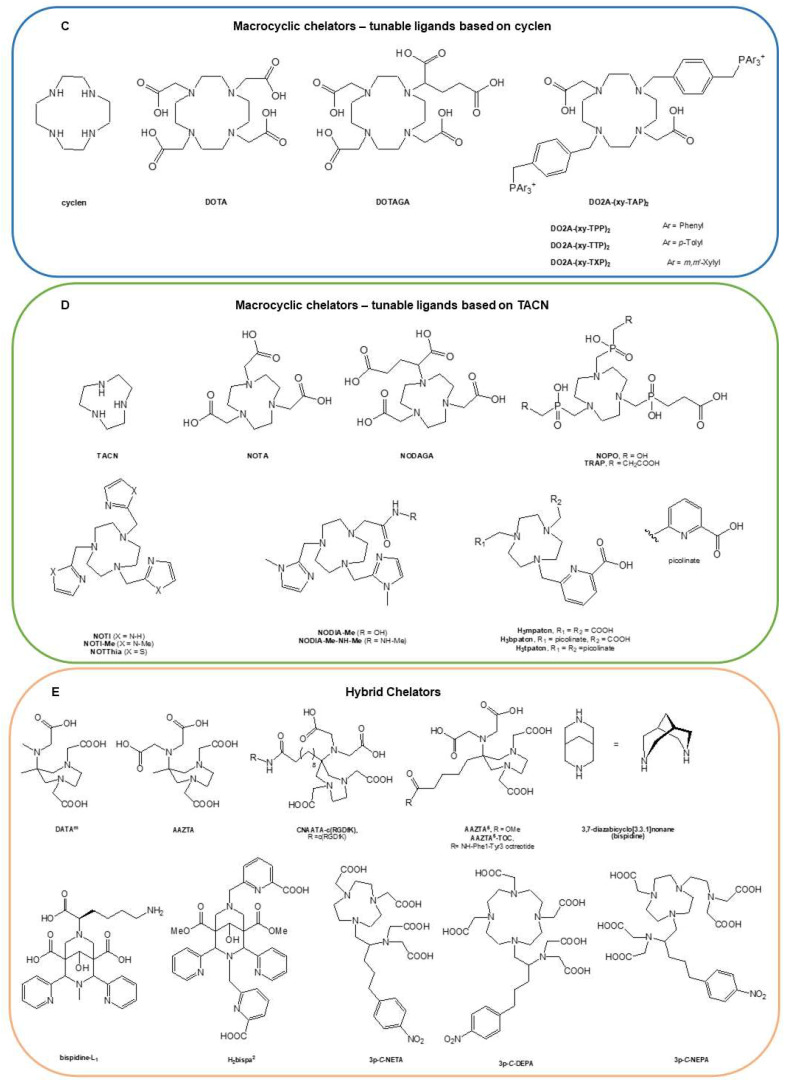
Radionuclide chelators: (**A**) Acyclic chelators, (**B**) macrocyclic chelators — based on D18C6, (**C**) macrocyclic chelators — based on cyclen, (**D**) macrocyclic chelators — based on TACN, and (**E**) hybrid chelators. Reproduced with permission from Elsevier publisher 80.

In another PSMA-617-based experiment, ^68^Ga-labelled derivatives whose 2-Nal region was swapped with 2-indanylglycine (Igl) or 3,3-diphenylalanine (Dip) did not exhibit substantial enhancements 101. Wüstemann *et al*. also investigated the effect of different chelators on the pharmacokinetics of PSMA-617 without modifying the core 102. When looking at tumour uptake and retention, CHX-A-DTPA conjugate performed best out of the eight chelators considered since the kidneys' ability to excrete and remove the drug was hampered. The chelators of conventional radionuclides are presented in **Figure 10**.

## The Emergence of Targeted Radiotheranostics: Then

The radiotracer concept, which underlies the use of radionuclides and radiopharmaceuticals to investigate the behaviour of stable atoms and molecules, was originally introduced by George de Hevesy, who is known as the "father of nuclear medicine." The "tracer principle" claims that minute quantities of radiopharmaceuticals can be used to explore the system and participate in biological processes without altering them 103. Despite its tremendous expansion, particularly during the past two decades, nuclear medicine has remained a relatively obscure subspecialty after more than 80 years of clinical medical history. Nuclear medicine has also pioneered the notion of "radiotheranostics", which combines therapy and diagnosis. The use of ^131^I for thyroid imaging and therapy is one of the first and most successful examples of this principle.

There is also a variety other, more recent, candidates which could be represented by exemplary [^68^Ga]Ga-PSMA-11 and [^177^Lu]Lu-PSMA-617. Remarkably, [^68^Ga]Ga-PSMA-11 PET/CT imaging has various detection sensitivities in the context of PCa, particularly for pelvic nodal metastases, where its sensitivity was recorded at 40% 104. Furthermore, another study quantified its sensitivity at 0.74, highlighting its potential in identifying PCa metastases 105. This imaging modality is distinguished by its improved diagnostic performance, offering similar sensitivity to alternative methods but with a threefold increase in positive predictive value for the detection of pelvic nodal metastasis. However, the efficacy of [^68^Ga]Ga-PSMA-11 is limited by the limited frequency of histopathological confirmation of the detected lesions 65. This limitation points to the necessity for additional research that integrates histopathological standards of truth, aiming to solidify the diagnostic accuracy and clinical applicability of [^68^Ga]Ga-PSMA-11 in the nuanced landscape of PCa management 104.

In a phase I study with 56 advancing mCRPC candidates, patients received up to five doses of [^177^Lu]Lu-PSMA-617 (the average local dosage per cycle was approximately 5.76 GBq; range, 3.6-8.8 GBq) with no observed severe side effects, demonstrating good tolerance 106. In a subsequent, single-arm phase II study, 50 males with progressing mCRPC and positive PSMA PET/CT results received an average of four cycles of [^177^Lu]Lu-PSMA-617 (the average local dosage per cycle was 7.5 GBq; range, 4-8 GBq) 107. At three months, 64% of patients with visceral and nodal metastasis achieved a complete or partial response according to the Response Evaluation in Solid Tumours (RECIST) 1.1. However, 13% of the patients experienced thrombocytopenia, which was the only grade 3 or 4 side effect. At the time of progression, patients with a primary response were subjected to further treatment with [^177^Lu]Lu-PSMA-617, and 73% of the patients had an unconfirmed decrease in PSA of at least 50%. All patients with mCRPC who received cabazitaxel were included in a randomised phase II study (TheraP) to compare [^177^Lu]Lu-PSMA-617 to conventional treatments 108. This study involved patients with either [^177^Lu]Lu-PSMA-617 or cabazitaxel treatment. Two-thirds of the candidates who were treated with [^177^Lu]Lu-PSMA-617 out of one-third who were treated with cabazitaxel had a PSA level that decreased to half or more than its original titre.

Additionally, the one-year progression-free survival (PFS) rates were approximately 19% and 3%, respectively. In general, the rates of grade 3 and 4 toxicities were 33% for men treated with [^177^Lu]Lu-PSMA-617 and 53% for men treated with cabazitaxel. Grade 3 and 4 neutropenia were less frequent with [^177^Lu]Lu-PSMA-617 than with cabazitaxel (4% vs. 13%), although a substantial reduction in thrombocyte counts was less frequent with cabazitaxel than with [^177^Lu]Lu-PSMA-617; 0% vs. 11%, respectively. Notably, in these investigations, most candidates in the trial who were treated with [^177^Lu]Lu-PSMA-617 reported significantly decreased discomfort 108.

In September 2021, the outcomes of the VISION phase III international, prospective, randomised, and landmark study were announced to the public 36. This study enrolled 831 patients with mCRPC who had PSMA positive lesions as confirmed by [^68^Ga]Ga-PSMA-11 PET/CT imaging. The control was defined as patients with at least one PSMA-positive metastatic lesion with no PSMA-negative lesions and after at least one androgen receptor pathway inhibitor treatment or one or more taxane regimens that worsened their condition. Two taxane regimens were given to approximately 39% and 43% of the [^177^Lu]Lu-PSMA-617 candidates, respectively. The participants were randomised to receive four cycles of 7.4 GBq [^177^Lu]Lu-PSMA-617 combined with standard of care (SOC) or SOC alone. ARPIs (e.g., enzalutamide and abiraterone), plus radiation therapy, denosumab, bisphosphonates, and glucocorticoids, are allowed treatments for SOC. Throughout the trial, patients were required to maintain a castrated testosterone level. Patients in the SOC group did not receive any immuno-, radio-, chemo-, or combined experimental therapies due to a lack of safety data. Six [^177^Lu]Lu-PSMA-617 dosages were permissible. The results revealed that patients who received [^177^Lu]Lu-PSMA-617 had longer overall survival (OS) and PFS than did those who received SOC alone (median PFS, 8.7 vs. 3.4 months; median OS, 15.3 vs. 11.3 months) regardless of the visceral distribution pattern, functional state, concurrent ARPI use, or age. The hazard ratio (HR) for OS among patients with severe liver metastases (n = 48) was approximately 0.87, with a 95% confidence interval (CI) of 0.53-1.43. The percentage of men with a verified PSA response (a considerable reduction in PSA of more than 50% from the baseline) was approximately 46%, with a SOC of [^177^Lu]Lu-PSMA-617. In comparison, only 7% of the associated toxicity was related to treatment with [^177^Lu]Lu-PSMA-617 (e.g., grade 1 or 2 xerostomia, leukopenia, thrombocytopenia, dry eyes, nausea, and vomiting). The incidences of grade 3 or 4 adverse events for bone marrow suppression, nausea and vomiting, and renal impairment were 23%, 1.5%, and 3.4%, respectively, compared to 7%, 0.5%, and 2.9%, for SOC alone. Based on these findings, the FDA designated [^177^Lu]Lu-PSMA-617 as a breakthrough treatment. This finding implies that this technique was quickly tested for use in men with PSMA-positive mCRPC whose cases have deteriorated following ARPI and chemotherapy 36.

Further trials are being conducted to examine the efficacy of [^177^Lu]Lu-PSMA-617 in various clinical settings and combination with other treatments. The possible synergy between ARPI and PSMA-TRNT is of great interest. PSMA expression increases in response to androgen deprivation therapy (ADT) and ARPI therapy. According to a preliminary investigation that examined biopsy samples of metastatic and primary PCa tissue from men before and after ADT, PSMA expression increased above the baseline value in all of the metastatic samples and half of the primary PCa samples 109.

In more recent prospective research, individuals with mCRPC who began treatment with an ARPI showed increased PSMA expression. Only a 15% median decrease in the PSA level was achieved for seven patients with mCRPC after receiving PET/CT scans with [^68^Ga]Ga-PSMA-11 at baseline before ARPI and on days 9, 18, and 28 after ARPI. Upon initiation of ADT males with mCRPC demonstrated a 45% median increase in the maximum standardised uptake value (SUVmax) on day 9, which plateaued by day 28, and a 15% median decrease in PSA 110. Given that androgen suppression causes an increase in PSMA expression, there is interest in combining PSMA-TRNT with ARPI therapy. This is because there is a chance that the two treatments will work better together 111. Recently, a randomised phase II trial (Enza-p [NCT04419402]) led by Louise Emmett and collaborators in Australia was designed to compare [^177^Lu]Lu-PSMA-617 with enzalutamide against enzalutamide in males with mCRPC to establish its effectiveness and safety 112.

In chemo-naïve mCRPC and mHSPC patients, as well as in combination with poly(ADP-ribosyl) polymerase (PARP) inhibition and programmed death 1 (PD-1)-based immunotherapy, additional trials are needed to evaluate the effectiveness of [^177^Lu]Lu-PSMA-617 in androgen-targeted treatment. By replacing ^177^Lu with the alpha emitter ^225^Ac, which has a higher LET and a shorter tissue penetration range, researchers are hoping to enhance the anticancer effectiveness of PSMA-617 27. Refractory or naïve patients with mCRPC to [^177^Lu]Lu-PSMA-617 were included in a recent prospective cohort trial to evaluate the effectiveness and safety of [^225^Ac]Ac-PSMA-617 113. Approximately 25% of the sensitive individuals and 39% of the insensitive patients had a 50% or greater decrease in PSA in response to [^177^Lu]Lu-PSMA-617. Initially, the sole side effect noted in clinical trials was xerostomia or mouth dryness. First patient studies with [^225^Ac]Ac-PSMA-617 revealed high uptake in tumour lesions with tumour/background ratios of 29.6±13.5 (SUV mean ratio) and 33.5±9.7 (SUV max ratio) 114. As of late 2017, [^225^Ac]Ac-PSMA-617 had been administered to 80 patients. Early findings from a July 2016 study highlighted outcomes for two patients with [^68^Ga]Ga-PSMA-11 PET/CT, confirming PSMA-positive lesions. Patients received a 100-kBq (3 µCi) dose of [^225^Ac]Ac-PSMA-617 per kilogram of body weight every two months. The results showed a reduction in PSA levels from more than 3,000 ng/mL to less than 0.1 ng/mL, extending initial life expectancy projections from less than four months to undetectable PSA levels 11. At the time of the study's publication, the follow-up period had reached 23 months, with some patients now being observed for more than four years 115. In South Africa, ground-breaking outcomes were reported in February 2022 from a cohort of 53 patients receiving [^225^Ac]Ac-PSMA-617 treatment. Based on the data, 91% of these patients experienced a reduction in PSA levels of more than 50%, demonstrating the potent efficacy of targeted alpha therapy (TAT). Notably, the median OS for patients who achieved a greater than 50% decrease in PSA levels was still not reached at the 55-month follow-up, underscoring the potential for extended survival 116. These findings illuminate the promising horizon of TAT in enhancing treatment paradigms for not only PCa, particularly in settings where conventional therapies have limited impact. Recent studies have also highlighted promising outcomes from combining lower doses of [^177^Lu]Lu-PSMA-617 with [^225^Ac]Ac-PSMA-617 in tandem therapy 117. This strategy seeks to optimise therapeutic outcomes and reduce adverse effects, providing a balanced and efficient treatment regimen. Despite its potential, further clinical trials and research are essential to confirm its effectiveness and safety and to optimise the dosage.

Currently, phase I trials using alpha emitters ([^225^Ac]Ac-PSMA-617 [NCT04597411] and [^225^Ac]Ac-huJ591 [NCT04946370]) are ongoing. In preclinical studies, simultaneously blocking programmed death-ligand 1 (PD-L1) and using radiotherapies that target PSMA were shown to be effective strategies 118. Pembrolizumab is being evaluated in phase I trials in combination with [^177^Lu]Lu-PSMA-617, [^225^Ac]Ac-huJ591, NCT03805594 and NCT04946370. In 2021, the first results of the [^177^Lu]Lu^-^PSMA-617 phase Ib experiment were reported at the regular congress of the European Society for Medical Oncology (ESMO). The initial findings indicated that this combination was well tolerated and potentially beneficial. While 27 of the 37 individuals had unverified PSA declines of more than 50%, seven of the nine patients had radiographic improvement 119. Identifying the relative benefits of such combination therapy is a major challenge, and additional controlled research is needed to address this issue. To improve the efficacy of ICI therapy, novel anti-PSMA therapies are being developed. REGN5678 is a bispecific antibody that targets both PSMA and CD28 120. With high hopes, a phase I/II trial (NCT03972657) is now being conducted on individuals with mCRPC who are being treated with REGN5678 (anti-PSMAxCD28) alone or in combination with cemiplimab (anti-PD-1).

### Improved Low-Molecular-Weight Inhibitors for PSMA Targeting

There is a critical need for an open-ended variety of ligands because of damage to healthy organs, despite the encouraging results of multiple clinical studies utilising low-molecular-weight inhibitors tagged with beta or alpha emitters for PSMA targeting. Therefore, improving the targeting mechanism *via* more effective ligands has become the focus of most preclinical trials.

### The Incorporation of the Albumin-binding Domain

To limit damage to healthy organs during PSMA-TRNT/-TAT, the dose of the radiolabelled ligand should be decreased; however, this would likely reduce the effectiveness of the anti-tumour agent. This issue could be resolved by extending the blood circulation duration of radiolabelled tracers, which would likely boost tumour uptake and thus enable the injection of a smaller amount of the radiolabelled tracer while maintaining the same level of tumour targeting 121. The circulation duration of rapidly eliminated compounds can be successfully extended by incorporating a plasma protein binding domain 122. Its high abundance and relatively long blood circulation time (the half-life of albumin is approximately 19 days) make albumin an appropriate plasma protein target 123. In addition to extending circulation time, the addition of an albumin-binding domain may also provide other advantages; for instance, the overexpression of albumin-binding proteins, such as SPARC and the glycoprotein 60 (gp60) receptor, which are essential for angiogenesis and capillary permeability, respectively, in tumour environments can lead to increased tumour uptake of albumin-conjugated tracers 124. Additionally, when the proportion of permeable to impermeable vasculature in diseased and healthy tissues increases, the radiolabelled ligand will concentrate in the tumour because of the larger size of the albumin-conjugated tracer 125. Several research teams improved the PSMA-targeting efficiency of small molecule inhibitor tracers *via* the attachment of several varieties of albumin-binding domains, for instance, 4-(*p*-iodophenyl) butyric acid 126. A range of PSMA albumin-binding tracers, for example, HTK01169, CTT1403, RPS-063, RPS-027, and DOTA-PSMA-ALB-02, have shown improved tumour uptake linked to an extension of blood circulation time. In contrast to the findings of Müller *et al*., and during *in vivo* murine tests, the inclusion of these albumin-binding domains significantly boosted both the absorption and retention of these tracers in the kidneys, which may have been induced through prolonged blood half-lives. Comparable findings were derived from the use of the PSMA-targeting conjugate DOTA-EB-MCG, in which the albumin-binding domain was fused to truncated Evans blue (tEB) 125.

The objective of subsequent trials was to capitalise on enhanced tumour uptake while simultaneously preventing boosted renal absorption and retention. By comparing 4-(*p*-iodophenyl) butyric acid with a *p*-(tolyl)-moiety as an albumin binder connected to PSMA-617 by a further lysine moiety, Umbricht *et al*. created two novel targeting molecules, PSMA-ALB-53 and PSMA-ALB-56. 127. The *p*-(tolyl)-moiety in PSMA-ALB-56 is a weaker albumin binder than that used with PSMA-ALB-53, and the ^177^Lu-labelled form was cleared more rapidly. Intriguingly, *in vivo* studies revealed that [^177^Lu]Lu-PSMA-ALB-56 had a significant survival advantage over [^177^Lu]Lu-PSMA-617, which was attributed to its superior tumour uptake and three larger tumour-to-kidney accumulation ratios. Despite the increased risk of renal damage, a weaker albumin binder that leads to more tumour uptake, owing to albumin binding, maybe the best balance. Several research groups have focused on producing homomultimeric tracers with multiple PSMA-binding domains to increase the binding affinity of PSMA-specific tracers. *In vitro*, these multivalent PSMA-specific tracers exhibited greater binding affinity, and *in vivo* tumour retention was enhanced. However, these tracers have not yet been utilised in the clinic for therapeutic purposes 25. In recent years, [^177^Lu]Lu-HTK03121 and [^177^Lu]Lu-HTK03123 demonstrated high peak uptake (104 ± 20.3 and 70.8 ± 23.7%ID/g, respectively) in LNCaP tumour xenografts and were sustained up to 120 h after injection 128. Dosimetry calculations showed that, compared with [^177^Lu]Lu-PSMA-617, [^177^Lu]Lu-HTK03121 and [^177^Lu]Lu-HTK03123 delivered 18.7- and 12.7-fold greater absorbed doses to the tumour but only 6.4- and 6.3-fold greater absorbed doses to the kidneys, leading to 2.9- and 2.0-fold improvements in the tumour-to-kidney absorbed dose ratios, respectively 128.

The development of [^177^Lu]Lu-EB-PSMA-617 as a radioligand integrates the PSMA-targeting capability with the attributes of Evans blue, which results in a high *in vitro* binding affinity to PSMA 129 with an IC_50_ value of 10.77 nM. This affinity is notably competitive with that observed for PSMA-617 130. SPECT imaging studies have confirmed the superior tumour uptake and retention characteristics of [^177^Lu]Lu-EB-PSMA-617 compared to [^177^Lu]Lu-PSMA-617, suggesting its potential effectiveness in PCa therapy. Further biodistribution assessments revealed a significantly elevated tumour uptake of [^177^Lu]Lu-EB-PSMA-617, quantified at 138.87 ± 26.53%ID/g, which markedly surpassed the uptake levels of [^177^Lu]Lu-PSMA-617 (4.28 ± 0.25%ID/g) 24 hours post-injection 130. In parallel, [^177^Lu]Lu-LNC1003 was synthesised by leveraging a PSMA-targeting framework coupled with Evans blue to create a novel radioligand. The binding affinity and specificity of PSMA were validated through cellular uptake and competitive binding assays in the 22Rv1 tumour model, which exhibited a moderate expression level of PSMA 131. The preclinical pharmacokinetics of [^177^Lu]Lu-LNC1003 were meticulously evaluated through SPECT/CT imaging and biodistribution studies in mice bearing 22Rv1 tumours. Additionally, radioligand therapy experiments were systematically conducted to explore the therapeutic impact of [^177^Lu]Lu-LNC1003, providing a comprehensive assessment of its potential efficacy in a preclinical setting 131.

These findings highlight the potential of albumin-binder derivatives for enhancing the efficacy of PSMA-targeted radiotherapy. Further clinical studies were also conducted to validate these promising preclinical results 128, 132, 133.

Chen's group developed two albumin binder-conjugated FAPI radioligands, TEFAPI-06 and TEFAPI-07 132. These ligands were derived from FAPI-04 and were optimised by conjugating two types of well-studied albumin binders. The binding affinities (Kd) of [^68^Ga]Ga-TEFAPI-06 and [^68^Ga]Ga-TEFAPI-07 for FAP were 10.16 ± 2.56 nM and 7.81 ± 2.28 nM, respectively, which are comparable to that of [^68^Ga]Ga-FAPI-04 132. Comparative PET imaging in HT-1080-FAP and HT-1080 tumour-bearing mice have shown the ability of these two tracers to target FAP *in vivo* 132.

The development of homomultimeric and heterodimeric PSMA tracers for PCa diagnosis and therapeutics aims to overcome the limitations of conventional tracers by enhancing binding affinity and tumour retention 134, 135. Homomultimeric tracers with multiple PSMA-binding domains have shown increased binding affinity and tumour retention in preclinical studies, yet their clinical application is pending. Heterodimeric ligands bind to other tumour marker targets, such as hepsin or αvβ3 integrin, suggesting the need for a more nuanced targeting approach but facing challenges in terms of binding efficacy and off-target uptake 136, 137. Despite not showing superior imaging properties, the PSMA/GRPR heterodimers have specific targeting capabilities. However, they also present challenges such as high uptake in non-target organs. A notable quantitative finding is the long activity retention of the [^125^I]BO530 heterodimer in PC3-PIP tumours, which has an unfavourable tumour-to-kidney ratio of 1.2 ± 0.3 at 24 hours post-injection, highlighting potential renal toxicity concerns 138. This research underscores the need for further investigations to optimise these innovative tracers for clinical use.

In a recent publication, Hensbergen *et al*. introduced an innovative second-generation ^99m^Tc-labelled tracer designed for enhanced imaging PSMA-expressing tumours. The novel aspect of this tracer lies in its incorporation of a fluorescent dye aimed at refining the tracer's photophysical attributes for improved diagnostic accuracy 139. The study meticulously quantified the brightness of these tracers, achieving a range from 0.3 to 1.5 × 10^4 M^-1 × cm^-1, indicative of the potential for heightened imaging resolution and sensitivity. A critical evaluation of the tracer's interaction with plasma proteins revealed a high level of serum binding, ranging from 85.0% ± 2.3% to 90.7% ± 1.3%, as did notable serum stability, with values between 76% ± 0% and 89% ± 6%. These parameters are essential for assessing the pharmacokinetic profile of a tracer and influence its distribution and efficacy *in vivo*. Furthermore, the affinity of these tracers for PSMA was assessed through the determination of half-maximal inhibitory concentration (IC_50_) values, which varied from 19.2 ± 5.8 nM to 175.3 ± 61.1 nM, revealing a spectrum of binding efficiencies that underscore the nuanced interaction between the tracer and the PSMA target. The *in vivo* performance of these tracers was rigorously analysed, revealing a wide range of tumour-to-prostate and tumour-to-muscle ratios (0.02 ± 0.00 to 154.73 ± 28.48 and 0.46 ± 0.28 to 5,157.50 ± 949.17, respectively) 139. Such metrics provide insight into tracer biodistribution and selective accumulation in tumour tissues over non-target tissues, a crucial factor for the specificity of PSMA-targeted imaging. Among the various tracer candidates explored, ^99m^Tc-EuK-(SO3)Cy5-mas3 stood out because of its high IC_50_ of 19.2 ± 5.8 nM and remarkable tumour-to-muscle ratio of 5,157.50 ± 949.17. These findings not only highlight the potent affinity of the tracer for PSMA but also highlight its superior ability to delineate prostate tumours with high contrast in imaging studies 139.

In conclusion, the addition of an albumin-binding domain to a PSMA-targeting radiotracer improved the circulation half-life and tumour absorption. On the other hand, the considerable increase in renal absorption and retention is undesirable, and the best practice, unless optimised, is to be avoided. Additionally, the longer half-life of these precisely designed tracers in the blood may cause more toxic effects in the salivary and lacrimal glands, bone marrow, and other organs and tissues, which entails more harm than benefits for the general well-being of patients.

### Terbium: A Novel Nuclear Medicine "Swiss Army Knife"

The radioisotopes of two lanthanide elements, Tb and Lu, have risen to prominence in the last 20 years. ^177^Lu is now routinely utilised in hospitals, while four terbium radioisotopes, terbium-149 (^149^Tb), terbium-152 (^152^Tb), terbium-155 (^155^Tb), and terbium-161 (^161^Tb), have taken centre stage and were revealed as some of the most potent radionuclides for therapy and diagnosis 140. Terbium research is currently gaining traction due to its four short-lived radioisotopes, ^149^Tb (alpha-PET), ^152^Tb (PET isotope), ^155^Tb (SPECT isotope), and ^161^Tb (beta minus and Auger emitter), which provide choices for all major nuclear medicine modalities, empowering theranostics using chemically similar compounds, all of which can be considered in one or more fields of nuclear medicine. Members of this new quadruplet family, which combines therapeutic and diagnostic radioisotopes, exhibit appealing nuclear properties and have the suggested capacity in nuclear medicine. However, the greatest challenge in using these radioisotopes is linked to sufficient production capacity 141.

Notably, for the first time, Frederik Cleeren and colleagues from KU Leuven and the Belgian Nuclear Research Centre (SCK. CEN) could label human serum albumin (HSA) with ^161^Tb. Utilising a range of commercially available bifunctional chelators and a lysine coupling method, they were able to successfully conjugate with p-SCN-3p-C-NETA, p-NCS-Bz-DOTA-GA, p-SCN-Bn-CHX-A"-DTPA, and p-SCN-Bn-DOTA. The radiochemical labelling yields for all HSA constructions using ^161^TbCl_3_ were more than 98% at ambient to moderate temperatures ranging from 25 °C to 40 °C. 142. Using these bifunctional ligands, the radiolabelled structures were shown to be stable for 24 hours in human serum at 37 °C.

In a study by Tschan *et al*., ^161^Tb-labelled albumin-binder-conjugated PSMA-targeting agents demonstrated high tumour uptake and an enhanced tumour-to-kidney absorbed dose ratio 143. Another study compared the dosimetry and therapeutic efficacy of ^161^Tb and ^177^Lu in tumour-bearing mice using SibuDAB and PSMA I&T 144. ^161^Tb decays with a similar half-life to ^177^Lu, but in addition to the emission of β^-^-particles and γ-rays, ^161^Tb also emits conversion and Auger electrons 144. This makes it particularly effective for eliminating micrometastases. The ^161^Tb-labelled ligands were found to be therapeutically more effective than their ^177^Lu-labelled counterparts. As a result of the albumin-binding properties, [^161^Tb]Tb/[^177^Lu]Lu-SibuDAB had an enhanced blood residence time and greater tumour uptake (62%-69% injected activity per gram at 24 h after injection) than did [^161^Tb]Tb/[^177^Lu]Lu-PSMA I&T (30%-35% injected activity per gram at 24 h after injection). [^161^Tb]Tb-SibuDAB inhibited tumour growth more effectively than [^161^Tb]Tb-PSMA I&T, as can be ascribed to its 4-fold increase in the absorbed tumour dose 123. Several novel PSMA-targeting agents labelled with other isotopes of Tb (such as ^149^Tb, ^152^Tb and ^155^Tb) are currently being evaluated in preclinical studies 145.

A study by the nuclear medicine team at King Hussein Cancer Center in Amman, Jordan, published the first-in-human SPECT/CT results following a well-tolerated dose of ^161^Tb-based PSMA-TRNT with no treatment-related adverse events 123. Two clinical trials of ^161^Tb-based PSMA-TRNT in PCa are currently underway and will provide valuable insights 123.

## Ongoing Clinical Trials: The Promise of Light

In light of the key clinical studies for PET imaging (LIGHTHOUSE, SPOTLIGHT, PSMA-PETgRT, PSMA-SRT, and PROSTAGE) (**Table 3**), the primary clinical trials for PET imaging (ProPSMA, PRIMARY, and LIGHTHOUSE), as well as the primary radioligand treatment studies for radioligand therapy (VISION, PSMAddition, PSMAfore, PRINCE, LuPSMA, LuPARP, and TheraP), we currently have a better grasp of the capabilities and limitations of PSMA theranostics. Elucidating the biology of PSMA expression, regulation, and function would aid in the development of novel sequential and combinatorial tactics for increasing response and safety 146, 147. In a retrospective case series, promising anti-cancer efficacy and minimal toxicity were described for the first time 148. According to a meta-analysis of 10 PSMA-TRNT trials, the PSA concentration was reduced by more than 50% in 51% (123/238) of patients with mCRPC treated with prior enzalutamide or abiraterone.

In the phase II TheraP trial, despite the anticancer activity of PSMA-TRNT, all patients developed recurrence 149. Among the putative mechanisms of acquired resistance are heterogeneity, loss of PSMA expression, and the inability to deliver a sustained fatal dosage to the target. Combining PSMA-targeted treatments with drugs that upregulate PSMA expression, increase tumour radiosensitivity, target specific PSMA-binding sites, or demonstrate complementary anticancer effects are potential techniques to improve PSMA-TRNT 150. Several possible combinations are being investigated for this purpose in ongoing clinical research. Some of these include combining PSMA-TRNT with AR-targeted treatments, DNA repair inhibitors, immunotherapies, chemotherapy, or other PSMA-TRNTs (**Table 4**).

## Clinical Consequences, Limitations, and Prospects: Future

Nearly four decades after the discovery of PSMA, researchers and physicians developed the first FDA-approved PSMA-targeted PET agents. This has made it easier to choose between local and systemic treatments for early illness and paved the way for developing promising novel PSMA-targeted radiotherapeutics for advanced disease stages. It is envisaged that PSMA-targeted diagnostic and therapeutic methods could be utilised in the early stages of mCRPC and mHSPC since PSMA is highly expressed in these phases and because TRNT is well tolerated.

PSMA-based imaging has various critical clinical implications. As a result of breakthroughs in the early yet exact diagnosis and localisation of persistent PCa, patients may be eligible for local treatment. These tandem approaches are the topic of ongoing investigations. Moreover, the use of PSMA-based PET/CT scans to discover novel, specific biomarkers is becoming increasingly indispensable. As mentioned in this report, the effectiveness of PSMA-TRNT depends on PSMA expression in the tumour. However, the innate heterogeneity of PSMA expression is a significant limitation, and PSMA expression downregulation is widespread in advanced mCRPC patients, primarily due to lineage plasticity 111.

Some anomalies in DNA repair mechanisms may lead to a higher level of biomarker expression, while patients with neuroendocrine transformation small-cell differentiation and visceral metastases have a lower level of PSMA expression. Approximately 15% of the people in the VISION study were not suitable candidates for treatment since they did not have a PSMA-dominant lesion and had metastases that did not express PSMA. Consequently, to identify each patient's eligibility for PSMA-directed therapy, it is standard practice to assess the expression status of their PSMA by employing PSMA PET/CT. The heterogeneity of PSMA expression has been used as a proxy for monitoring therapy response in detailed research on circulating tumour cells (CTCs); in addition, some investigations consider liquid biopsy diagnostic tests to ascertain the heterogeneity of PSMA 151.

The reliance on these materials on tumour PSMA expression most conspicuously limits PSMA-directed theranostics. In addition, PSMA expression has been shown to exhibit high inter- and intraindividual variation. For instance, a specific PSMA PET/CT investigation of prostate biopsies with positive PSMA and further negative PSMA foci was performed for 100% of HSPCs and 84% of mCRPCs 152. In addition, additional biomarkers and clinical predictors need to be developed for the use of PSMA-targeted theranostics in SOC. This approach enables the screening of intrinsically resistant patients and partial or complete loss of biomarker expression, as well as the anticipation of an increase in neuroendocrine PCa or androgen receptor ambivalence.

It is possible to combine PSMA-TRNT with other modalities because of its elevated tolerance. In preclinical investigations, PSMA radionuclide therapy and immunotherapy have synergistic effects, and clinical studies are now systematically considering such conclusions. Since PSMA expression is linked to DNA damage and abnormalities, the use of PARP inhibitors and PSMA-based combination therapy appears prudent. Given the gradual prevalence of PSMA-negative tumours, combining radiopharmaceuticals or immunotherapies targeting somatostatin receptors is ideally suited for treating neuroendocrine-differentiated PCa antigens 153.

After the progression of ARPI treatment and taxane chemotherapy for mCRPC patients with PSMA-positive PET/CT, the data indicate that [^177^Lu]Lu-PSMA-617 should be added to the SOC following FDA approval. However, even though this is a very major accomplishment, it is important to understand its limitations. It is reasonable to administer [^177^Lu]Lu-PSMA-617 to men who are not eligible for or previously treated with taxane. Following the progression of ARPI therapy, both the SPLASH (NCT04647526) and PSMAfore (NCT04689828) studies aimed to assess the effectiveness of both [^177^Lu]Lu-PSMA I&T and [^177^Lu]Lu-PSMA-617, respectively. Intriguingly, compared to that of the higher-energy beta emitter (^90^Y), the beta emission of ^177^Lu in large tumour masses has shown diminished effectiveness 35. Although well tolerated, patients with bone marrow metastases showed an inadequate response to the use of [^177^Lu]Lu-PSMA-617. Such inadequacy could be overcome by the application of the rather experimental agent [^225^Ac]Ac-PSMA-617; nonetheless, the latter has been associated with dose-limiting xerostomia 154.

As PSMA-targeted drugs gain popularity in the clinic, disease resistance will unavoidably emerge as a secondary concern. Within or between disease sites, PSMA-negative foci may be resistant to treatment and develop into dominant clones. Neuroendocrine and small-cell differentiation may occur from the evolutionary forces exerted by PSMA-TRNT. Alteration of the PSMA protein at the molecular level within the body might also generate resistance mechanisms. For instance, some of its splice variants are only cytosolic and lack a transmembrane domain. However, the lack of a cytoplasmic tail prevents the internalisation of PSMA in preclinical studies 155. Researchers will constantly be seeking new approaches to combat resistance and identify at-risk patients alongside the continued development of PSMA-targeted radiotheranostics.

## Improvement of Targeted Radionuclide Therapy and Diagnosis

**Figure 11:**
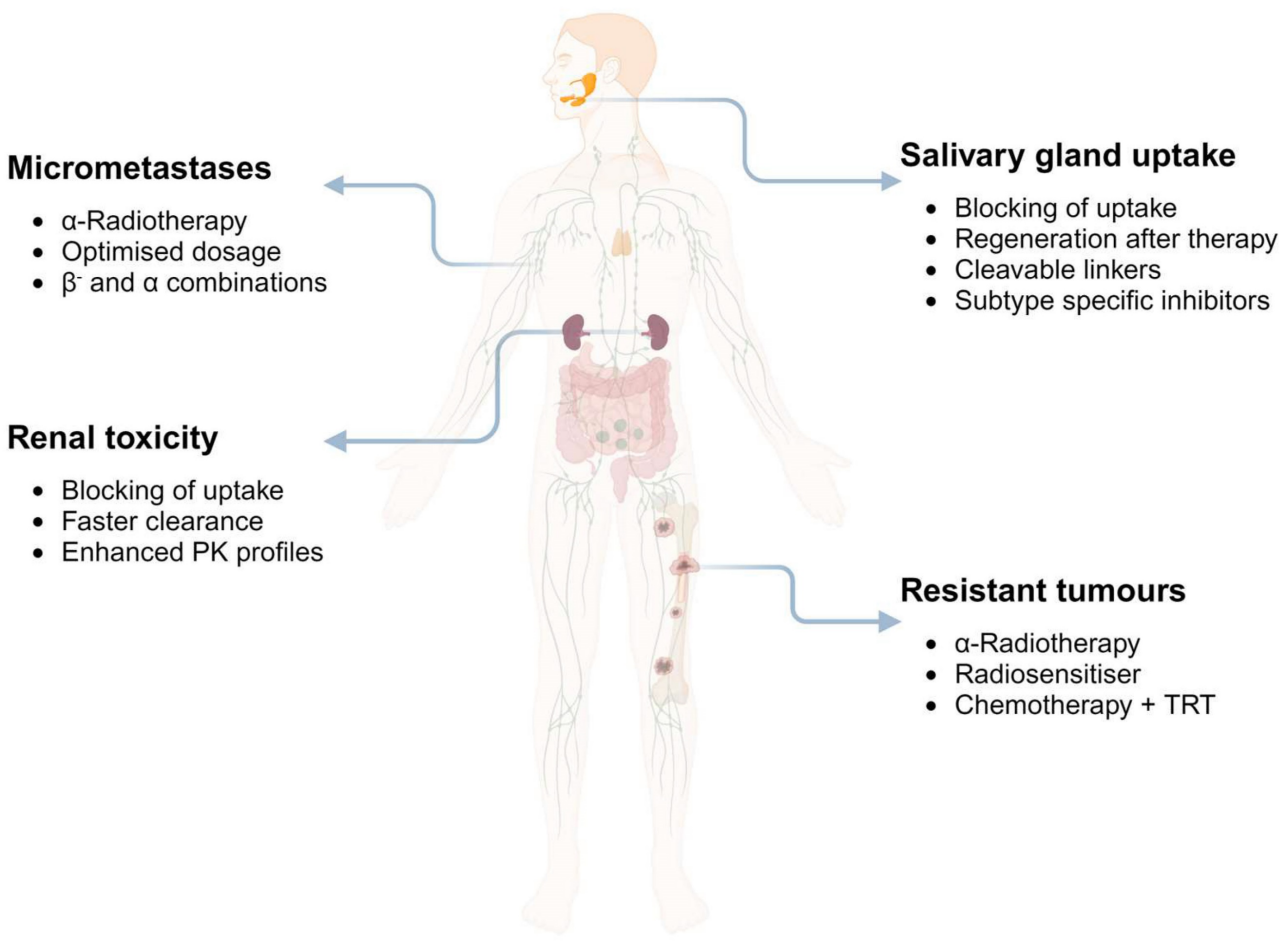
Illustration of the main challenges faced by clinical PSMA-TRNT and possible answers to them.

In addition to the therapeutic effects previously discussed, improvements in therapeutic efficacy based on structural changes to medications and combination therapy methods could lead to more choices for increasing the TRNT in PCa patients. Because clinical issues such as limited therapeutic effectiveness or serious adverse effects continue to occur during PSMA-TRNT (**Figure 11**), novel therapy alternatives may be of significant benefit to patients.

## Combinatorial PSMA-targeted Theranostics

In clinical trials, combinations of radiotherapy and chemotherapy have already shown superior efficacy over single therapies 156. Due to the novelty of PSMA-related TRNT, few in-depth combinatorial investigations have been performed. Engaging complementary radiosensitizing agents (e.g., hypoxic cell sensitisers) to augment tumour radiosensitivity is one technique for enhancing the efficacy of TRNT. Cytotoxic medicines improve the susceptibility of cancer cells to DNA damage, resulting in enhanced tumour elimination via TRNTs. Although most of these trials were conducted in the context of external beam radiation, the results illustrate the potential of this combination strategy 157. Using LNCaP cells cultured as multicellular tumour spheroids, Mathias Tesson and his colleagues evaluated the impact of combining various cytotoxic agents (e.g., the inhibitors of PARP-1, topoisomerase-I, proteasome, and MDM2-P53) with the radiopharmaceutical drug [^131^I]MIP-1095; the authors found that all the drugs remarkably inhibited the growth of the spheroids 158. In separate studies, 32 men with mCRPC were administered six cycles of [^177^Lu]Lu-PSMA-617 (the average local dosage per cycle was approximately 7.5 GBq) on day one, while increasing the dose of the radiosensitizing drug idronoxil (which induces tumour cell apoptosis by activating the mitochondrial caspase system, inhibiting X-linked inhibitor of apoptosis (XIAP), and disrupting FLICE inhibitory protein (FLIP) expression) on days 1-10 of a six-week cycle. The findings of the combined effect showed that 91% of individuals had measurable PSA responses, and the median survival time was 17.1 months 159.

In contrast, the combination of mitotic inhibitors and antimicrotubular chemotherapeutic drugs (taxanes) is still the most explored combination. For instance, in the first trial to evaluate taxane radiosensitisation in PCa, docetaxel was concurrently administered in combination with three-dimensional conformal radiation therapy; this combination resulted in moderate to severe acute and late toxicity in PCa cells 160. Marcus Kelly and his colleagues in Australia were the first to examine the promising combination modality of ^177^Lu-radiolabelled monoclonal antibody (anti-Lewis Y mAb hu3S193) with docetaxel or the epidermal growth factor tyrosine kinase inhibitor AG1478. Their research revealed that [^177^Lu]Lu-hu3S193 induced considerable cytotoxicity and apoptosis *in vitro* and could significantly inhibit PCa development *in vivo* in a dose- and specificity-dependent manner 161. Similarly, in a clinical trial that was established to evaluate the safety, dose-limiting toxicity, and maximum tolerated dosage of ^177^Lu-labelled anti-PSMA huJ591 (NCT00916123), mCRPC patients were given docetaxel (75 mg/m^2^) every three weeks, along with two fractionated doses of [^177^Lu]Lu-huJ591 (the average local dosage was 1.48 GBq/m^2,^ with a maximum of 2.96 GBq/m^2^). The combination of [^177^Lu]Lu-huJ591 delivered as a single fractionated cycle with docetaxel and prednisone was found to be effective in individuals with mCRPC. Furthermore, there was no DLT at any dosage level without pre-selection for PSMA; 73.3% of patients had a >50% decrease in PSA, while 78.6% had favourable CTC counts; precise targeting of known disease locations was observed, as was a high preliminary effectiveness signal 162. Another randomised phase II clinical study was conducted with a focus on patients with de novo mHNPC to study the activity and safety of [^177^Lu]Lu-PSMA-617 (UpFrontPSMA [NCT04343885]). This trial compared the administration of a radiopharmaceutical agent administered sequentially with docetaxel versus the administration of docetaxel alone 163. Furthermore, in a case study, Maharaj *et al*. highlighted how [^177^Lu]Lu-hu3S193 treatment with Taxol-based chemotherapy as a radiosensitiser benefitted a patient with mCRPC. After three years, the patient received eight cycles of ^177^Lu]Lu-hu3S193 at a total dosage of 51.8 GBq. All therapy was effective and was generally well tolerated. There was a great response to re-challenge with low-dose docetaxel, and no tumour resistance was observed 164.

In an innovative phase I clinical trial spearheaded by researchers from the University of California, San Francisco, a novel therapeutic strategy was examined for its potential to treat mCRPC 146. This approach entailed the combination of [^177^Lu]Lu-PSMA-617 radioligand therapy with pembrolizumab immunotherapy, which involved the administration of a single priming dose of [^177^Lu]Lu-PSMA-617 followed by subsequent pembrolizumab treatment. The outcomes of this pioneering trial were encouraging, as the mCRPC patient cohort demonstrated significant anti-tumour activity and minimal toxicity. Notably, enduring responses in a subset of patients were characterised by an increase in the presence of circulating T cells and a notable decrease in the activity of immunosuppressive cells after receiving the priming dose 146. Further exploration of combination therapies was highlighted in a review published in the Journal of Nuclear Medicine. This review shed light on the tolerability and effectiveness of PSMA-TRNT, despite acknowledging the existence of patient subsets with either inherent or acquired resistance to such treatments. Addressing this challenge, the scientific community is actively pursuing novel TRNT combinations that incorporate innovative hormonal agents, PARP inhibitors, and immunotherapies. These combinations aim to overcome resistance, thereby enhancing both the efficacy and safety of mCRPC treatments 165. Additionally, the synergistic effects of radiopharmaceuticals and immunotherapy have attracted significant amounts of attention within the research domain. Emerging evidence underscores the immunostimulatory effects of radiopharmaceuticals, particularly spotlighting the combination of [^177^Lu]Lu-PSMA-617 with immune checkpoint inhibitors as a viable and promising strategy for managing late-stage PCa 166. This combined approach marks a significant leap forward in the ongoing quest to offer more effective and personalised treatment options for patients with this formidable disease, potentially setting a new benchmark in the therapeutic landscape of PCa.

To summarise, combination therapeutic strategies offer significant untapped potential, and additional studies are needed. Combining TNRT with chemotherapeutics, immunotherapy, or external beam radiation may improve survival in PCa patients.

## Theranostic Applications of Radio-conjugated Nanomaterials

**Figure 12:**
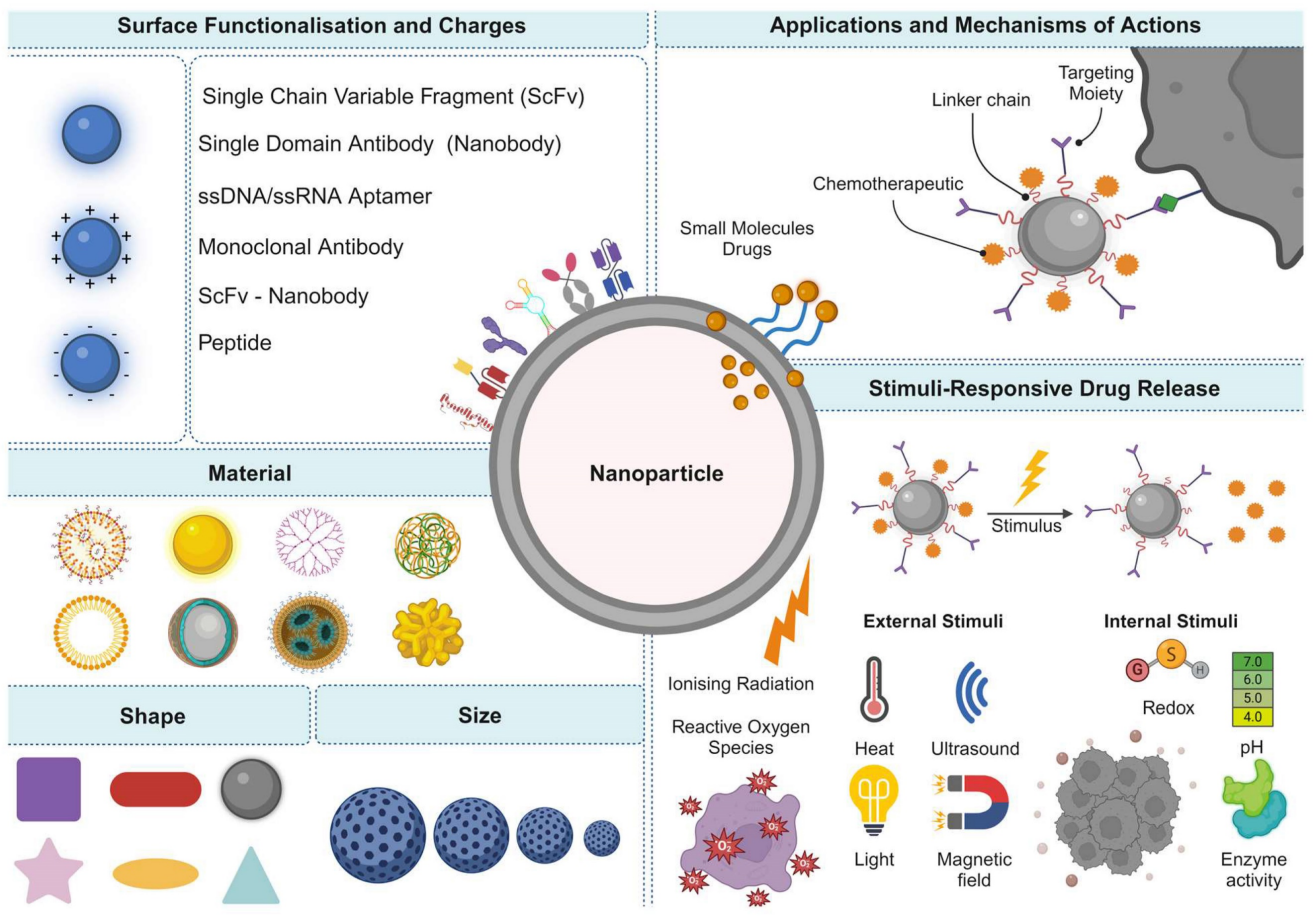
Unlocking the potential of nanoparticles for cancer therapy: A comprehensive overview of surface functionalisation techniques, responsive stimuli, and modes of action. The figure showcases the versatility of nanoparticles, highlighting the diverse materials, shapes, and sizes that can be employed. Additionally, the illustration highlights the various surface functionalization techniques and responsive stimuli mechanisms, presenting a comprehensive understanding of the potential applications of nanoparticles in cancer therapy.

Recent advances in nanotechnology have led to the development of new nanomaterials that may be useful in cancer therapy. Nanostructures, which can be created artificially or naturally and have important inorganic properties, such as carbon lattices, polymers, metals, or silica, are structures on the nanometre size scale (often less than 100 nm); on a biological basis; on a lactic acid, dextran, or lipid-based basis; or as substances with a sugar or lipid structure 167. The ability of nanomaterials to be functionalized with particular ligands, leading to nanoscale, tailored carriers for imaging and therapeutic compounds, is one of the fascinating features of this class of materials 168. **Figure 12** shows a spectrum of nanomaterials, each uniquely suited for application in theranostics.

SPECT/PET imaging and TRNT/TAT are just a few of the many theranostic applications made possible by coupling functionalized nanomaterials with certain radionuclides. In research investigating the theranostic use of radiolabelled nanoparticles for PCa, gamma-emitting radionuclides have been used to track *in vivo* biodistribution and measure the targeted delivery of nanostructure-based therapies using SPECT imaging. A potential theranostic technique for PCa that expresses PSMA was described in a recent study by Yari *et al*. This technique relies on premade liposomes to which a PSMA-targeting lipopolymer has been bonded 169. Yari and his group developed a liposome-based theranostic delivery system targeting PSMA^+^ LNCaP prostate cancer cells. By synthesising a lipopolymer (P^3^) composed of a PSMA ligand (PSMAL), PEG_2000_, and palmitate and incorporating it into preformed liposomes, P^3^-loaded liposomes were created. Compared with plain liposomes, these liposomes loaded with doxorubicin and radiolabelled with ^99m^Tc demonstrated a more than threefold increase in uptake by LNCaP cells. This specificity was further confirmed by the >3-fold increase in the delivery of doxorubicin to LNCaP cells. *In vitro* cytotoxicity assays revealed that doxorubicin-loaded P^3^-loaded liposomes were significantly more toxic to LNCaP cells than to control cells, reducing the IC_50_ value by approximately fivefold without affecting PSMA-negative PC3 cells. This work highlights the potential of PSMAL-functionalized liposomes for specific and effective PCa theranostic delivery 169. Despite the widespread use of docetaxel in cancer therapy, its non-selective toxicity underscores the urgency of enhanced delivery techniques. Recent advancements have focused on leveraging nanocarriers and targeting agents to improve the solubility, stability, and tumour specificity of drugs. However, each method presents unique challenges, from the need for functionalization in PEGs to solubility issues in chitosan and control difficulties in mesoporous silica nanoparticles. Gold nanoparticles (AuNPs) have emerged as promising yet underexplored carriers due to their biocompatibility and functional flexibility. The integration of dendrimers and AuNPs showcases the potential for improved drug delivery, despite commercial reproducibility concerns. Clinical translation of these innovations remains limited, with formulations such as BIND-014 targeting PSMA in phase I trials, illustrating the gap between laboratory success and clinical application. The future of docetaxel therapy lies in overcoming these hurdles through continued research, with nanotechnology-driven vectorization offering a path toward more effective, targeted, and tolerable cancer treatments 170.

Nonetheless, nanomaterials have a wide range of therapeutic applications in PCa, including radiosensitisation, targeted medicine, and immunotherapy. Plasmonic nanoparticles, namely, gold nanoparticles (AuNPs), can trigger irreversible photothermal cellular destruction by generating localised heat when exposed to light; this process is known as localised surface plasmon hyperthermia 171. A bombesin (BN) analogue-functionalized ^99m^Tc/^177^Lu-AuNP-based theranostic radiopharmaceutical for PCa was developed by Jiménez-Mancilla *et al*. and is suitable for both plasmonic photothermal treatment after laser irradiation and targeted radiotherapy 172. Silva *et al*. extended the investigations of Gd^3+^ and ^67^Ga for MRI and SPECT, respectively, by using DOTA-linked targeted AuNPs to bind metal ions. When combining SPECT/PET imaging with radionuclide therapy for gastrin-releasing peptide receptor (GRPR)-positive PCa, ^68^Ga, ^90^Y, ^177^Lu, or erbium-165 (^165^Er) have been proposed as alternatives to ^67^Ga 173.

Moeendarbari *et al*. described an additional prospective theranostic application based on AuNPs. They generated injectable brachytherapy nanoseeds by fusing the radiopharmaceutical agent ^103^Pd onto an approximately 120 nm hollow gold nanoshell. The preservation of nanoseeds following direct injection into a PCa tumour xenograft was tracked using SPECT/CT due to the low-energy X-ray emission of palladium-103 (^103^Pd), while the therapeutic efficacy was evaluated using a parallel [^18^F]FDG PET study 174. Various PSMA-targeted nanosystems, such as self-assembled nanoparticles (NPs), liposomal structures, water-soluble polymers, dendrimers, and other macromolecules, are under development for PCa radiotheranostics 175. Notably, the groups of Deng *et al*. and Meher *et al*. have made substantial contributions to this field. Deng *et al*. developed functionalized PSMA-chlorin e6 (PSMA-Ce6) nanoparticles to enhance photodynamic therapy for PCa. PSMA-Ce6 self-assembled into nanoparticles with a hydrophobic core and a hydrophilic shell, which significantly enhanced the accumulation of PSMA-Ce6 in the tumour 176. They linked the hydrophobic photosensitiser Ce6 with the small hydrophilic molecule PSMA ligand through covalent bonding to form the functionalized PSMA-Ce6. In the aqueous phase, PSMA-Ce6 self-assembled into nanoparticles with a hydrophobic core and a hydrophilic shell, which significantly enhanced the accumulation of PSMA-Ce6 in the tumour 176. Meher *et al*. discussed the significance and up-to-date development of “PSMA-targeted nanocarrier systems for radioligand imaging and therapy of PCa”². PSMA-targeted nanosystems, such as self-assembled nanoparticles, liposomal structures, water-soluble polymers, dendrimers, and other macromolecules, are under development for PCa theranostics due to their multifunctional sensing and therapeutic capabilities 175. Furthermore, a multifunctional melanin-like polydopamine (PDA) nanocarrier decorated with a low-molecular weight PSMA inhibitor was prepared 177. This nanocarrier was used for ultrasound-guided combined photodynamic/photothermal therapy (PDT/PTT) of PCa. Positive pharmacokinetics, renal clearance profiles, and low off-target localisations were found in preclinical *ex vivo* bioavailability, *in vivo* PET imaging, and dosimetry studies, suggesting that these nanoparticles may be useful theranostic tools for a variety of applications in the management of PCa, from nuclear medicine imaging and image-guided surgery to TRNT and TAT 178. Using ^18^F-labelled fluoroazomycin-arabinoside ([^18^F]FAZA)-PET imaging, Xiang *et al*. quantified the oxygenation achieved by nanoscale perfluoro-carbon droplets to study the indirect therapeutic potential of nanomaterials in a xenograft mouse model of a hypoxic human PCa model 179. The radioconjugation of beta particle-emitting radionuclides, such as ^177^Lu, with oxygenating nanoemulsions may enhance the therapeutic benefits of these reactive oxygen species (ROS) while allowing direct imaging of the administered nanomaterial distribution using SPECT. These exemplary preclinical experiments highlighted the theranostic potential of nanomaterials with radionuclide conjugation. The authors emphasised the importance of additional research to gather preliminary clinical experiences in this newly emerging field of study.

Despite this growth, no nanoparticle theranostics have been produced to adequately address therapeutic demands. All currently available nanoplatforms have various challenges. Long-term toxicity concerns, the price of gold nanoparticles, their difficulty in biodegrading, and the bulky structure of certain nanoparticles are just a few of these issues. It is crucial to demonstrate the advantages and synergy of this combined strategy, in addition to carrying out and proving the nanoscale integration of imaging and therapeutic activities. Theoretically, a theranostic agent based on NPs might enhance diagnostic and therapeutic response monitoring by delivering drugs to a diseased location while simultaneously capitalising on its imaging role. Potential long-term safety difficulties associated with these nanoparticles, especially non-biodegradable nanoparticles that may persist inside the body for a longer period following administration, are the major fundamental hurdles to the clinical application of nanomaterials. Despite a mountain of evidence demonstrating the short-term safety of nanoparticles, their long-term toxicity has yet to be properly studied. Furthermore, the interactions of these cells with the immune system are poorly understood. Consequently, additional comprehensive research is needed to assess all the clinical safety measures used in these patients.

## Side Effect Minimisation

The administration of highly cytotoxic radioactive nuclides is intrinsic to the use of radiopharmaceuticals. These chemicals can cause substantial damage to healthy tissue if they accumulate in large quantities. The kidneys and salivary glands are the main cumulative off-target organs affected by PSMA-targeting drugs. This is why the focus is now on reducing the off-target uptake of radiopharmaceuticals in these tissues. Despite its great promise, PSMA-TRNT/TAT must balance survival, disease-related symptoms, and direct adverse consequences. Xerostomia is likely the most common complication and represents the main limitation of these approaches. Symptoms vary depending on the absorbed dose and the isotope used, but symptoms are notably relevant for TAT using low-molecular-weight ligands. Radiolabelled PSMA-targeting antibodies do not generally accumulate in salivary glands 37; different molecular weights and specific ionic charges of PSMA radioligands have been hypothesised to be potential factors for this phenomenon 180. To a certain extent, the minimal concentration of targeting ligands in the salivary glands supports nonspecific absorption 181. Research studies by Rupp and Tonnesmann on [^68^Ga]Ga-PSMA-11 and [^177^Lu]Lu-PSMA-617, respectively, provided more evidence in favour of this hypothesis 36. Various trials conducted using [^177^Lu]Lu-PSMA-617 therapy have demonstrated only mild to severe symptoms following its administration 182; Kratochwil *et al*. reported that although [^225^Ac]Ac-PSMA-617 was effective, severe xerostomia was reported as a common side effect and a dose-limiting agent 183.

To address this issue, numerous methods of shielding the salivary glands have been evaluated in clinical investigations. Sialendoscopy is another therapeutic option based on evidence from thyroid cancer patients with radioiodine-induced sialadenitis 184. Rathke *et al*. investigated the impacts of saline irrigation and steroid injection on the salivary glands of 11 patients before and after each cycle of [^225^Ac]Ac-PSMA-617. Although promising effects on salivary gland function were observed, xerostomia did not manifest after multiple cycles of [^225^Ac]Ac-PSMA-617 185. In a preliminary attempt, external cooling of the salivary glands was anticipated to reduce PSMA inhibitor uptake due to vasoconstriction 186. When ice packs were applied to one parotid gland one hour before and four hours after application, side-by-side comparisons indicated no significant differences. Baum *et al*. injected botulinum toxin into the parenchyma to restrict off-target uptake and inhibit gland metabolism 187. In a study conducted on humans for the first time, parotid gland uptake was reduced by up to 64% relative to baseline. To prevent off-target nonspecific binding, attempts were made to block salivary glands with non-radiolabelled PSMA inhibitors such as 2-PMPA 188, PSMA-11 79, or TrisPOC-2-PMPA 189. Significant reductions in tracer uptake were observed in the kidneys and salivary glands. Specifically, 2-PMPA doses of 0.2-1 mg/kg appear optimal for sustaining nearly complete tumour uptake while simultaneously achieving near-total blockade of specific renal PSMA binding 44. Similarly, a recent study by Harsini *et al*. revealed that monosodium glutamate (MSG) significantly decreased the uptake of the salivary gland, kidney, and other normal-organ PSMA radiotracers in human subjects 188. The effects of MSG on PSMA tracer absorption were similar to those of cold chemical blockade, which caused a corresponding reduction in tumour uptake, which may limit the benefits of this approach for diagnostic and therapeutic applications 188. Furthermore, as Paganelli *et al*. recently showed, oral administration of high doses of folic acid (5 and 10 mg) was predicted to lead to a decrease in [^68^Ga]Ga-PSMA-11 salivary gland and kidney uptake, while intake of folate-containing food or vitamin supplements had no relevant effects 44, 190. Sarnelli *et al*. investigated the idea of protecting salivary glands against polyglutamate by combining it with sugar mannitol. However, the clinical data did not show any substantial changes in PSMA-targeting inhibitor accumulation in either organ 191. Ultimately, the current precautions are insufficient, and additional improvements are desperately needed. The regeneration of salivary glands with stem cells after radiation exposure 192 might be a conceivable solution to this issue. However, additional research is needed to improve patient care and prevent post-treatment morbidity, particularly as TAT haas gained popularity. The recent exploration of renal toxicity associated with PSMA-TRNT in patients with mCRPC by the Eiber group has shed vital light on a pressing concern. Through a meticulous retrospective analysis of the estimated glomerular filtration rate (eGFR) over a year post PSMA-TRNT, they revealed that a considerable segment of patients faced moderate to severe reductions in eGFR 12 months after beginning their therapy. Alarmingly, 45% of the patients experienced at least moderate eGFR decreases, with nearly half of this group witnessing severe or very severe downturns 193. The study revealed that a greater number of initial risk factors was associated with a greater decrease in the eGFR, raising concerns about the renal implications of such treatments. These insights not only highlight the renal dangers inherent in PSMA-TRNT but also underscore the critical need for vigilant renal function monitoring in these patients. Despite the therapeutic promise, the current gaps in long-term nephrotoxicity data underscore the urgent need for further prospective studies. Such research is crucial for a more holistic understanding of the nephrotoxic potential of PSMA-TRNT treatments.

## Conclusions and Future Outlook

Theranostics is the epitome of focused, individualised cancer diagnosis and therapy in the present era of personalised medicine since we can only treat what we can see. The molecular profile of each tumour is distinct, as should its treatment strategy. Radiotheranostics enables imaging of these specific tumour markers as well as patient selection and categorisation to identify the most effective treatment. Future research on theranostics can provide additional innovative therapeutic targets and enhance pharmacokinetics. In nuclear medicine, TRNT will become routine practice, and a more individualised approach to treatment in the future will be carried out by easing the transition from standard empiric doses to individualised treatment doses and cycles.

PSMA is a protein that is overexpressed in PCa cells. Imaging techniques that use PSMA as a target, such as PSMA PET/CT, are highly effective at detecting and locating PCa, even in the early stages of the disease. This approach can lead to earlier and more accurate diagnoses, which in turn can improve treatment outcomes for patients by allowing for more targeted, less invasive therapies. Additionally, PSMA-based imaging can also be used to monitor the effectiveness of treatment and detect recurrent disease. PSMA expression in PCa is highly variable, and PSMA can be absent in certain metastases. The factors that affect the clinical response to PSMA-TRNT have not been identified. In addition, how PSMA expression may be modulated for therapeutic reasons and how successful therapy combinations might be established are unclear. A better understanding of the biology underlying the use of PSMA could aid in the development of radiolabelled theranostics and other PSMA-based therapies. In recent years, PSMA-TRNT has emerged as a viable alternative to traditional mCRPC therapy regimens. This quick success has had a large effect on research into radiopharmaceuticals and has pushed large pharmaceutical companies to put more effort into nuclear medicine. With early data suggesting that PSMA-based TRNT may extend life expectancy more than competing therapeutic strategies, there has been much discussion about its role in the context of metastatic PCa and whether clinical outcomes may be improved by using TRNT at an earlier stage, such as in early mCRPC before hormone therapies or chemotherapy, or in mHSPC. There is compelling evidence for the efficacy of multiple combination approaches in mHSPC, and it is worthwhile to study the utility of implementing TRNT early in the PCa spectrum.

Multiple retrospective trials and preliminary prospective research have shown that PSMA therapeutics hold tremendous promise for treating advanced PCa. The PSMA trials paved the way for subsequent randomised controlled trials, such as TheraP and VISION. A clinical trial at Radboud University (NCT03828838) has begun enrolling patients with a modest disease burden who have not yet developed CRPC. Due to the extraordinarily high radioligand absorption by small lesions, TRNT may be more effective for treating low-volume disease. Researchers in Australia are designing a PSMA study for men with high-volume mHSPC as part of a PCa research partnership cofounded by the Movember Foundation, Cancer Australia, and the United States Department of Defense (UpFront PSMA trial). Additionally, [^177^Lu]Lu-PSMA-617 was examined in a neoadjuvant study of males with high-risk localised PCa before radical prostatectomy (RP) (LuTectomy trial). Moreover, various PSMA-targeting radiopharmaceuticals, including mAbs and mAb-derived structures, as well as low-molecular-weight agents, have been produced because of related research. After clinical translation, low-molecular-weight PSMA inhibitors appear to have a favourable molecular structure for TRNT and TAT. The present phase III results for [^177^Lu]Lu-PSMA-617 impressively demonstrate the substantial potential of low-molecular-weight TRNT for mCRPC, constituting the most favourable conditions for regulatory approval. FDA approvals of [^68^Ga]Ga-PSMA-11 and [^18^F]-DCFPyL provided additional support and paved the way for the approval of [^177^Lu]Lu-PSMA-617, which was approved in the US in March 2022 and in Europe in December 2022. Studies using alpha-emitters conjugated to PSMA-targeted ligands have shown promising results, especially in beta-resisting lesions; nonetheless, their high salivary gland absorption and accumulation in the kidneys continue to be the most significant limitations in the field of PSMA-TRNT.

Moreover, while the most widely used radioligands exhibit immense potential, other options and modifications are in development. By coupling PSMA-617 to the albumin-binding Evans Blue, a Chinese team recently demonstrated an increase in tumour formation and ligand retention. Several phase I trials (NCT03403595 and NCT03780075) are now recruiting participants to evaluate this technically upgraded ligand in larger cohorts. Another new small-molecule ligand, [^177^Lu]Lu-PSMA-R2, is being evaluated in phase I with mCRPC patients as part of the PROter project (NCT03490838).

Finally, ongoing and upcoming research is critical for enhancing the accuracy and efficacy of PCa treatment. This includes assessing current techniques, re-evaluating current strategies, finding knowledge gaps, and creating new ones. Such initiatives can result in improved patient outcomes and resource use. The benefits of low-molecular-weight inhibitors showed that PCa studies should focus on this type of drug. Additionally, issues such as unintended uptake in nontarget organs, disease relapse due to micrometastases, tumour heterogeneity, and resistance must be addressed by developing combined therapeutic approaches and introducing long-awaited alpha emitters into organised clinical settings. Significant areas of interest in preclinical PSMA research include improving existing PSMA-targeting tracers and novel linkers, developing hybrid ligands, experimenting with different radionuclides, improving therapeutic effects with the inclusion of radiosensitisers, and searching for selective compounds that block the accumulation of PSMA in healthy organs. The preclinical trials described in this review and the preclinical research that is still ongoing will affect what will happen in the clinic with PSMA-TRNT. In conclusion, PSMA radiotheranostics have achieved remarkable milestones in PCa diagnosis and treatment. To advance these therapies, efforts are focused on improving efficiency, reducing side effects, and addressing radionuclide stability and availability for PCa applications. The integration of therapeutic radionuclides into nanoparticles shows great potential, offering hope for improved outcomes and enhanced quality of life in PCa patients.

## Figures and Tables

**Table 1:** β^-^Emitters used in nuclear medicine and their basic properties 92.

Radionuclide	Half-Life	Emission	E_β(max)_/Range (Max)
^166^Ho	26.8 h	β^-^	1850 keV/9 mm
^153^Sm	46.3 h	β^-^	810 keV/3 mm
^67^Cu	61.9 h	β^-^/γ	575 keV/2.1 mm
^90^Y	64.1 h	β^-^	2284 keV/11.3 mm
^177^Lu	6.7 d	β^-^/γ	497 keV/1.8 mm
^161^Tb	6.9 d	β^-^/Auger/CE	150 keV/0.1 mm
^131^I	8.0 d	β^-^/γ	606 keV/2.1 mm
^89^Sr	50.5 d	β^-^	1491 keV/7.0 mm

**Table 2:** Basic properties of the α-emitters used in nuclear medicine 92, 194.

Radionuclide	Half-life	Emission	E_α(max)_/Range (Max)
^213^Bi	45.6 min	α/β^-^	8.32 MeV/84 µm
^149^Tb	4.1 h	α/β+	3.97 MeV/28 µm
^211^At	7.2 h	α	6.79 MeV/60 µm
^212^Pb	10.6 h	β^-^ to α ^212^Bi	6.05 MeV/80 µm
^225^Ac	10.0 d	α/β-	6.83 MeV/61 µm
^223^Ra	11.4 d	α	5.64 MeV/45 µm
^227^Th	18.7 d	α	6.14 MeV/100 µm

**Table 3:** Key prospective clinical trials of PSMA-TRNT in prostate cancer 146.

Parameter	Trial	Setting	Treatment	Phase	Primary endpoint	Outcome
Neoadjuvant	NCT04430192 (Lutectomy)	Localised or locoregional advanced prostate cancer with a high PSMA uptake and high-risk	^177^Lu-PSMA-617 (1-2 cycles)	Phase I/II single-arm	Absorption of radiation dosage in the prostate and lymph nodes	Active
	NCT04297410	Locally advanced prostate cancer with PSMA uptake	^177^Lu-PSMA I&T	Feasibility	Surgical safety; surgical histology; postoperative PSA	Recruiting
mHSPC	NCT04443062 (Bullseye)	HS oligometastatic PCs with a high PSMA uptake	^177^Lu-PSMA I&T vs SOC	Phase II randomised	Disease progression at six months	Recruiting (SOC= deferred androgen deprivation therapy)
	NCT04343885 (UpFrontPSMA)	Recent diagnosis of high-volume, hormone-free prostate cancer metastasis	^177^Lu-PSMA-617 followed by docetaxel vs docetaxel	Phase II randomised	Undetectable PSA rate at 12 months	Recruiting
	NCT04720157 (PSMAddition)	mHSPC	^177^Lu-PSMA-617 + NAAT vs. NAAT	Phase III	rPFS	Recruiting
mCRPC	NCT03042468	mCRPC (n = 44) with prior taxane treatment and at least 1 prior NAAT line	^177^Lu-PSMA-617 2 weeks apart	Phase I/II single-arm	DLT, MTD, R2PD	There was no DLT at any preplanned dose; RP2D: 22.2 GBq/cycle
	ANZCTR 12615000912583	mCRPC (n = 50; 30 in the initial phase, 20 in the expansion phase); at least one prior line of taxane chemotherapy	^177^Lu-PSMA-617	Phase II single-arm	% patients with ≥50% PSA decline	≥50% PSA decline: 64%
	NCT03392428 (TheraP)	mCRPC (n = 200) for which cabazitaxel was deemed the best therapeutic option; prior therapy with NAAT was permitted.	^177^Lu-PSMA-617 vs. cabazitaxel	Phase II randomised	% patients with ≥50% PSA decline	50% reduction of PSA from baseline (66% vs. 37%) in favour of ^177^Lu-PSMA-617, and P<0.0001.
	NCT03511664 (VISION)	mCRPC (*n* = 831); prior taxane chemotherapy and NAAT	^177^Lu-PSMA-617 + SOC vs. SOC (2:1)	Phase III randomised	OS, rPFS	OS: (15.3 vs. 11.3 months) favouring ^177^Lu-PSMA-617; HR: 0.62, and *P* < 0.001.
	NCT04689828 (PSMAfore)	mCRPC (*n* = 495); prior NAAT	^177^Lu-PSMA-617 vs. abiraterone or enzalutamide (2:1)	Phase III randomised	rPFS	Recruiting
	NCT04647526 (SPLASH)	Except in the case of HSPC, mCRPC with PSMA PET-positive illness; no prior NAAT or treatment.	^177^Lu-PSMA I&T vs. enzalutamide or abiraterone (2:1)	Phase III randomised	rPFS	Recruiting
	NCT03537391 (PROSTAGE)	Novel Imaging in Staging of Primary Prostate Cancer. Imaging for Prostate Cancer Metastasis Detection - Traditional Imaging (Bone Scan and CT) Versus PSMA-PET-CT, SPECT-CT and Whole-Body MRI.	Compare the diagnostic accuracy of ^18^ F-PSMA-1007 versus traditional imaging modalities in high-risk prostate cancer patients at the time of initial staging.		Despite the risk of false positive bone lesions, 18F-PSMA-1007 PET-CT outperformed all other imaging methods studied for the detection of primary distant metastasis in high-risk PCa.	Completed
	NCT03582774 (PSMA-SRT)	Evaluate the success rate of salvage radiation therapy (SRT) for recurrence of prostate cancer after prostatectomy with and without planning based on ^68^Ga-PSMA-11 PET/CT	Arm I: Receives SOC SRT without 68Ga-PSMA-11 PET/CT imaging. Arm II: Undergoes ^68^Ga-PSMA-11 PET/CT) imaging prior to receiving SRT.	Phase III randomised	The trial primarily focuses on the success rate of SRT, measured as biochemical progression-free survival after the initiation of SRT.	In progress, not accepting new patients as of the last update in July 2023. Estimated Completion Date: July 2025
	NCT03525288 (PSMA-PETgRT)	Compare BCR free survival radiotherapy informed by between PSMA-PET and conventional imaging only in men with high risk, recurrent, or oligometastatic prostate cancer	PSMA -PET/CT simulation	Phase II/III	Include acute and delayed toxicities, rate of failure, survival, health-related quality of life, and detection yield of PSMA PET imaging	Active, not recruiting as of last update in September 2023. Failure-free survival over 5 years

DLT = dose-limiting toxicity; HR = hazard ratio; HSPC = hormone sensitivity; MTD = maximum tolerated dose; NAAT = novel anti-androgen treatment; PET = positron emission tomography; rPFS = radiological progression-free survival; RP2D = recommended phase II dose; SOC = standard of care.

**Table 4:** Principal PSMA-TRNT combination trials in prostate cancer 146.

Combination strategy	Trial	Setting	Treatment	Phase
TRNT plus an immune checkpoint inhibitor	NCT03805594	mCRPC; PSMA PET-positive at three or more metastatic locations; prior treatment with NAAT; no prior chemotherapy, even in the setting of HSPC; no prior radiotherapy.	^177^Lu-PSMA-617 and pembrolizumab	1
	NCT03658447 (PRINCE)	mCRPC; prior therapy with NAAT; authorised prior docetaxel	^177^Lu-PSMA-617 and pembrolizumab	1/2
TRNT plus radiosensitiser	NCT03511664 (VISION)/(LuPIN)	mCRPC; prior treatment with taxane and NAAT	^177^Lu-PSMA-617 and idronoxil	1/2
TRNT plus PARP inhibitor	NCT03874884 (LuPARP)	previous treatment with NAAT plus taxane chemotherapy for metastatic colorectal cancer	^177^Lu-PSMA-617 plus Olaparib	1
TRNT plus novel antiandrogen therapy	NCT04419402 (ENZA-p)	mCRPC with PSMA-positive illness; no prior treatment other than in the setting of HSPC.	^177^Lu-PSMA-617 plus enzalutamide vs. enzalutamide	2

HSPC = hormone-sensitive prostate cancer; NAAT = novel anti-androgen treatment

## Abbreviations

ALS: Amyotrophic lateral sclerosis
ARPI: androgen receptor pathway inhibitor
CDC: Centres for Disease Control and Prevention
CRPC: Castration-Resistant Prostate Cancer
CTC: Circulating Tumour Cells
DCIBzL: 2-(3,4-dichlorobenzyl)indazole
DOTA: 1,4,7,10-tetraazacyclododecane-1,4,7,10-tetraacetic acid
DOTAGA: 2,2′,2”-(10-(2,6-dioxotetrahydro-2H-pyran-3-yl)-1,4,7,10-tetraazacyclododecane-1,4,7-triyl)triacetic acid
EMA: European Medicines Agency
ESMO: European Society for Medical Oncology
FALS: Familial ALS
FDA: U.S. Food and Drug Administration
FOLH1: Folate Hydrolase 1
GCPII: Glutamate Carboxypeptidase II
GMP: Good Manufacturing Practice
hARV: Human androgen receptor variant
HBED-CC: *N*,*N*'-Bis[2-hydroxy-5-(carboxyethyl)benzyl]ethylenediamine-*N*,*N*'-diacetic acid
HSPC: Hormone-sensitive prostate cancer
ICI: Immune checkpoint inhibitor
LET: linear energy transfer
mAbs: Monoclonal antibodies
mCRPC: Metastatic Castration-Resistant Prostate Cancer
MN: Motor Neuron
MRI: magnetic resonance imaging
NAAG: *N*-acetylaspartylglutamate
NAALADase: *N*-Acetylated Alpha-Linked Acidic Dipeptidase
NPs: Nanoparticles
PARP: Poly (ADP-Ribose) Polymerase
PCa: Prostate Cancer
PD-1: Programmed Death 1
PET: positron emission tomography
PET/CT: positron emission tomography/computed tomography
PET/MRI: positron emission tomography/magnetic resonance imaging
PFS: progression-free survival
PSA: prostate-specific antigen
PSMA: Prostate-Specific Membrane Antigen
RECIST 1.1: Response Evaluation Criteria in Solid Tumours version 1.1
SOC: Standard of Care
SPECT: Single-Photon Emission Computed Tomography
SPECT/CT: single-photon emission computed tomography/computed tomography
SUVmax): Maximum standardised uptake value
TAT: Targeted Alpha Therapy
TRNT: Targeted radionuclide therapy
2-PMPA: 2-phosphonomethyl pentanedioic acid
